# A Heat‐Inducible Rubisco Activase Safeguards Photosynthesis via Multilevel Regulation and Dual Function

**DOI:** 10.1111/pbi.70774

**Published:** 2026-10-20

**Authors:** Yuqing Cao, Xiaomian Zhang, Fangqian Qu, Mengxuan Liu, Zheng Li, Bing Liu, Xianbin Gu, Qianyi Hu, Hong Zhou, Liangsheng Zhang, Yiping Xia, Xiuyun Wang

**Affiliations:** ^1^ Genomics and Genetic Engineering Laboratory of Ornamental Plants, College of Agriculture and Biotechnology Zhejiang University Hangzhou People's Republic of China; ^2^ Department of Plant Pathology Washington State University Pullman USA; ^3^ Institute of Horticulture Zhejiang Academy of Agricultural Sciences Hangzhou People's Republic of China

**Keywords:** heat stress, photosynthesis, Rubisco activase

## Abstract

Rubisco activase (RCA) is critical for maintaining photosynthetic efficiency under heat stress, yet its regulatory mechanisms and functional diversification remain poorly understood. Here, we characterize a novel heat‐inducible RCA family member (RCA1), which plays a pivotal role in mediating multidimensional thermotolerance in a tropical azalea. Both heterologous and homologous overexpression of *RCA1* significantly mitigated heat‐induced cellular damage and better maintained photosynthetic capacity, confirming its essential function in thermal resilience. Heat‐induced *RCA1* expression is transcriptionally activated by a synergistic HSFA1‐HSFA2 complex and post‐transcriptionally fine‐tuned by extensive alternative splicing, forming a multilevel regulatory network under heat stress. Beyond its canonical Rubisco‐activating enzymatic activity, RCA1 exhibits an additional thermoprotective function that suppresses the thermal aggregation of several chloroplast proteins, such as Lhca2, PetC and PsaK. RCA1 also interacts with the constitutive RCA2, likely through heteromeric RCA complex assembly, which helps retain RCA2 in a soluble pool under heat stress. Evolutionary analyses reveal functional divergence within the RCA family, with convergent evolution of heat‐inducible *RCA* clades in Panicoideae grasses. Collectively, our findings uncover a novel dual‐functional heat‐inducible RCA, which provides a promising target for photosynthetic bioengineering to enhance crop thermotolerance and ensure agricultural sustainability under global warming.

## 1 Introduction

Heat stress represents one of the most damaging abiotic stresses that severely impact plant growth and development by disrupting critical cellular processes including membrane integrity, protein synthesis, and enzyme activity (Jagadish et al. 2021; Li et al. 2023). Among various physiological processes, photosynthesis exhibits particular sensitivity to elevated temperatures, with the Calvin cycle enzyme ribulose‐1,5‐bisphosphate carboxylase/oxygenase (Rubisco), the central catalyst of carbon fixation, experiencing significant activity reduction under heat stress, leading to substantial declines in photosynthetic efficiency (Busch and Sage 2017). Notably, while Rubisco itself demonstrates relative heat insensitivity, its essential regulatory protein Rubisco activase (RCA) shows marked heat instability under high temperature conditions (Crafts‐Brandner and Salvucci 2000; Sage et al. 2008). As a crucial AAA+ chaperone, RCA maintains Rubisco activity by removing inhibitory sugar phosphates from the catalytic site (Portis 2003; Bracher et al. 2017). Consequently, heat‐induced impairment of RCA function severely limits Rubisco activation capacity, ultimately reducing CO_2_ assimilation rates and compromising overall photosynthetic performance (Crafts‐Brandner and Salvucci 2000; Salvucci and Crafts‐Brandner 2004). This heat intolerance of RCA represents a critical bottleneck in plant thermotolerance and photosynthetic maintenance under heat stress.

In higher plants, Rubisco activase (RCA) usually exists as two principal isoforms: the longer α‐isoform (~46 kDa) with a C‐terminal extension and the shorter β‐isoform (~43 kDa) (Zhang et al. 2002; Wachter et al. 2013). These isoforms arise through different molecular mechanisms across species: in Arabidopsis ( *Arabidopsis thaliana* ), spinach ( *Spinacia oleracea* ), and rice ( *Oryza sativa* ), alternative splicing of a single pre‐mRNA generates both forms (Werneke et al. 1989; To et al. 1999); conversely, in species such as maize ( *Zea mays* ) and cotton ( *Gossypium hirsutum* ), the two isoforms are encoded by separate, distinct genes (Salvucci et al. 2003; Yin et al. 2014). Functionally, the α‐isoform often exhibits higher thermostability and its expression in species such as cotton and maize is induced under heat stress (Crafts‐Brandner and Salvucci 2002; Salvucci et al. 2003; Salvucci and Crafts‐Brandner 2004; Kurek et al. 2007; Ristic et al. 2009); however, exceptions exist, such as the thermotolerant TaRca1‐β isoform in wheat (Scafaro et al. 2019). These species‐specific adaptations underscore the importance of a thermostable activase under thermal challenge. Beyond the canonical role in activating Rubisco, growing evidence points to potential non‐catalytic functions of the heat‐induced *RCA* members in response to high temperature (Stainbrook et al. 2024), which have yet to be defined.

Given the robust heat‐inducible nature of these specialized RCA isoforms, identifying the molecular mechanisms that govern their rapid up‐regulation is essential for understanding plant thermal adaptation. Heat shock transcription factors (HSFs) serve as crucial regulators in the general plant heat response (Ohama et al. 2017). Under heat stress, activated HSFs recognize and bind to conserved heat shock elements (HSE, 5′‐nGAAnnTTCn‐3′) in the promoter regions of heat shock protein (*HSP*) genes, triggering rapid accumulation of these molecular chaperones that prevent protein denaturation and facilitate refolding of damaged proteins (Jacob et al. 2017; Ohama et al. 2017). Among the three classes of plant HSFs (A, B and C), HSFA1 members function as master regulators of the heat shock response transcriptional network (Scharf et al. 2012). Their activation involves HSP70/HSP90‐mediated release from inhibition under elevated temperatures, enabling them to orchestrate the expression of downstream *HSF*s including *HSFA2*, *HSFA3* and *HSFA7a* (Yoshida et al. 2011; Friedrich et al. 2021; Ren et al. 2024). Notably, HSFA2 emerges as the most strongly heat stress‐induced transcription factor, governing the expression of diverse *HSP*s and other heat shock response‐related genes such as galactinol synthase 1 (*GolS1*) and ascorbate peroxidase 2 (*APX2*) (Nishizawa et al. 2008; Shekhawat et al. 2022; Ma et al. 2024). While the regulatory roles of HSFs in orchestrating heat shock protein networks are well established, whether they directly participate in modulating core photosynthetic components, such as *RCA*, remains an open and intriguing question.

To address these knowledge gaps regarding how heat‐inducible RCAs are transcriptionally regulated and what non‐canonical functions they perform, we investigated *Rhododendron hainanense*, a woody plant endemic to the thermally variable montane forests of China's Hainan Island that exhibits remarkable high‐temperature adaptation (Zhao et al. 2018). Our previous multi‐omics work identified RCA1 as a key thermotolerance regulator in this azalea (Wang et al. 2020). In the present study, we provide a comprehensive multi‐level analysis to decipher the regulatory network and biological roles of RCA1. First, we analyse the superior heat induction and thermostability of the *RCA1‐X1* splice variant. Second, we test the hypothesis that the HSFA1‐HSFA2 complex serves as an upstream regulator, acting as a thermosensitive switch to control *RCA1* expression. Third, we characterize a novel thermoprotective chaperone function of RCA1 that goes beyond its canonical activase activity. Finally, we explore the evolutionary conservation of this regulatory module across photosynthetic organisms. Collectively, our findings uncover a multidimensional adaptive strategy of RCA1 in response to heat stress, offering valuable insights and potential targets for enhancing thermal resilience and safeguarding photosynthesis in crops under future climate scenarios.

## 2 Results

### 2.1 RCA1 Is Heat‐Inducible, With Its Splice Variant X1 Exhibiting a Distinct Advantage in Thermoinduction and Thermostability

To elucidate the functional divergence of the *RCA* gene family in response to heat stress in *R. hainanense* , we first analysed the molecular characteristics of its three members, *RCA1*, *RCA2*, and *RCA3*. Multiple sequence alignment (Figure S1A) and phylogenetic analysis (Figure S1B) revealed that the three share a relatively conserved ATPase domain. RCA1 and RCA2 share higher sequence similarity and a closer phylogenetic relationship, and they form a sister clade with RCA3. The transcriptome data (Figure S1C) showed that *RCA2* was the most highly expressed family member at 25°C, whereas *RCA1* and *RCA3* showed much lower expression. However, *RCA1* is markedly up‐regulated by heat (Figure 1A), nearly reaching the constitutive expression level of *RCA2*, at both 37°C and 42°C (Figure S1C). In contrast, *RCA2* and *RCA3* were mildly suppressed under heat treatments (Figures 1A and S1C).

**FIGURE 1:**
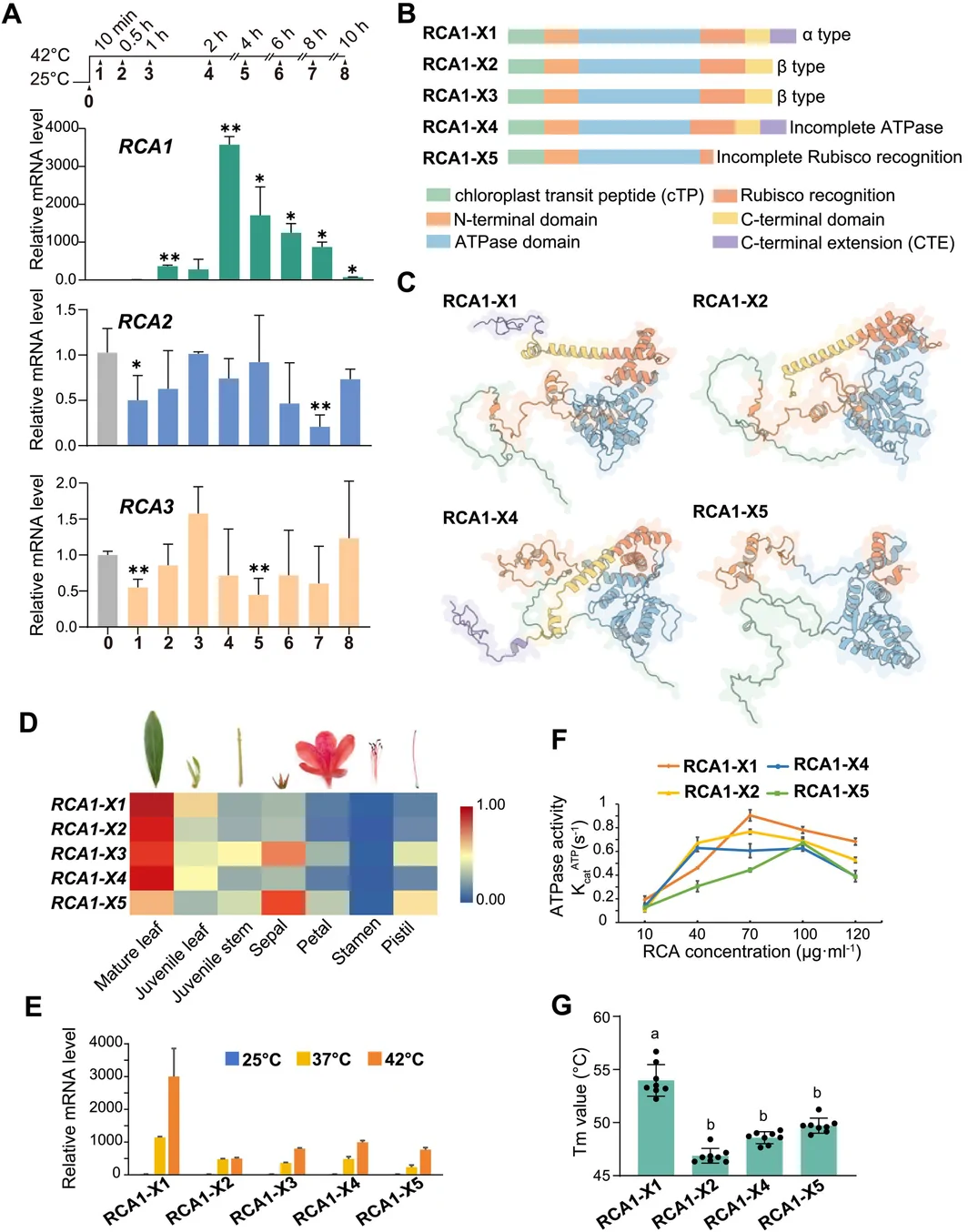
Functional characterization of RCA1 and its splice variants in *R. hainanense* . (A) Expression profiles of *RCA1*, *RCA2* and *RCA3* in leaves under sustained heat stress (42°C for 0 to 10 h). Heat treatment was initiated at 9:00 AM (Zeitgeber Time 1). The qRT‐PCR primer pair targeted a sequence shared by all splice variants for each gene. Data were normalized to *18S rRNA* and expressed relative to 0 h. Statistical significance was determined by Student's *t*‐test (*p < 0.05, **p < 0.01). (B) Domain organization of five RCA1 splice variants. (C) Predicted tertiary structures of monomeric RCA1 isoforms generated by AlphaFold3 and visualized in PyMOL using semi‐transparent surface models to reveal the internal secondary structures. The colour coding strictly corresponds to the functional domains defined in panel (B). (D) Tissue‐specific expression of *RCA1* variants in different organs of *R. hainanense* after a 3‐h heat treatment at 37°C. (E) Expression patterns of *RCA1* variants exposed to different temperatures (25°C, 37°C and 42°C) for 1 h. (F) ATPase activity of four recombinant RCA1 isoforms measured across a range of RCA concentrations (μg mL^−1^) using a continuous spectrophotometric coupled‐enzyme assay. The *y*‐axis *k*_*cat*_^ATP^ (s^−1^) denotes the catalytic turnover number per active site (number of ATP molecules hydrolysed per RCA1 active site per second). (G) Thermostability analysis of RCA1 isoforms, with *T*_*m*_ determined by DSF (*n* = 8 independent technical replicates per isoform; individual data points are shown). Values with different letters are significantly different according to a one‐way ANOVA followed by Duncan's test (*p* < 0.05). Data are the means ± SD (*n* = 3 for A, D, E, F; *n* = 8 for G).

Full‐length transcriptome analysis revealed that *RCA1* undergoes alternative splicing, generating multiple splice variants (Figure S1D). X1 contains the complete domain of the α isoform type, X2 and X3 have the domain of the β isoform type, while X4 and X5 have domain deletions to varying degrees (Figures 1B and S1E). Because *RCA1‐X2* and *RCA1‐X3* differ only in the 3′ UTR and encode the same protein, *RCA1‐X2* was used as the representative in subsequent protein‐level and transgenic assays. Three‐dimensional structure prediction (monomeric models, Figure 1C) also reveals differences among the splice isoforms. Subcellular localization shows that they all localize to chloroplasts (Figure S2A).

To further analyse the differences among different alternative splicing transcripts, the expression level analysis of *RCA1‐X1*–*X5* under different conditions was carried out. The results indicate that these transcripts are mainly expressed in green tissues, especially mature leaves, under heat treatment (Figure 1D). *X1* showed higher expression levels than other transcripts under both 37°C and 42°C (Figure 1E). Light also slightly induced *RCA1* transcript expression, but to a much lesser extent than heat treatment (Figure S2B).

Biochemical assays were further performed to elucidate the functional divergence among the isoforms at the protein level with recombinant proteins (Figure S2D–G). Among the RCA1 splice isoforms, X1 maintained significantly higher ATPase activity (Figure 1F) and exhibited the highest thermal stability, as indicated by its significantly elevated melting temperature (*T*_*m*_) in differential scanning fluorimetry (DSF) assays (Figure 1G; representative thermal denaturation curves are shown in Figure S2C). Together, these results suggest that the superior thermoinduction and thermostability of X1 underpin its functional advantage during thermal stress.

### 2.2 RCA1 Alleviates Heat‐Induced Damage in Arabidopsis and *R. hainanense*

To validate the role of RCA1 isoforms in mitigating heat stress, we generated heterologous transgenic complementation (*rca*/RCA1‐X1/2/4/5) using the leaky Arabidopsis *rca* allele (SALK_003204) and overexpression (WT/RCA1‐X1/2/4/5) lines in Col‐0 background, both driven by the constitutive CaMV 35S promoter. The successful generation of these lines was confirmed by genomic DNA PCR and qRT‐PCR, verifying T‐DNA integration and elevated *RCA1* transcript levels, respectively (Figures S3 and S4).

Under heat stress, transgenic complementation and overexpression lines exhibited significantly alleviated heat sensitivity regardless of the specific isoform, whereas *rca* mutants exhibited pronounced heat‐induced leaf damage (Figure 2A). In addition to alleviating leaf injury, *RCA1* expression also protected inflorescence development from high‐temperature inhibition (Figure 2B). Physiological analyses further demonstrated that although heat treatment inevitably caused a degree of photooxidative damage to photosystem II (PSII) across all genotypes (as reflected by the overall decline in *F*_*v*_/*F*_*m*_), transgenic plants maintained significantly better photosynthetic performance under these stress conditions. This was evidenced by lower relative electrical conductivity (REC), as well as higher *F*_*v*_/*F*_*m*_ and chlorophyll content compared with WT or *rca* mutants (Figures 2C,D and S5).

**FIGURE 2:**
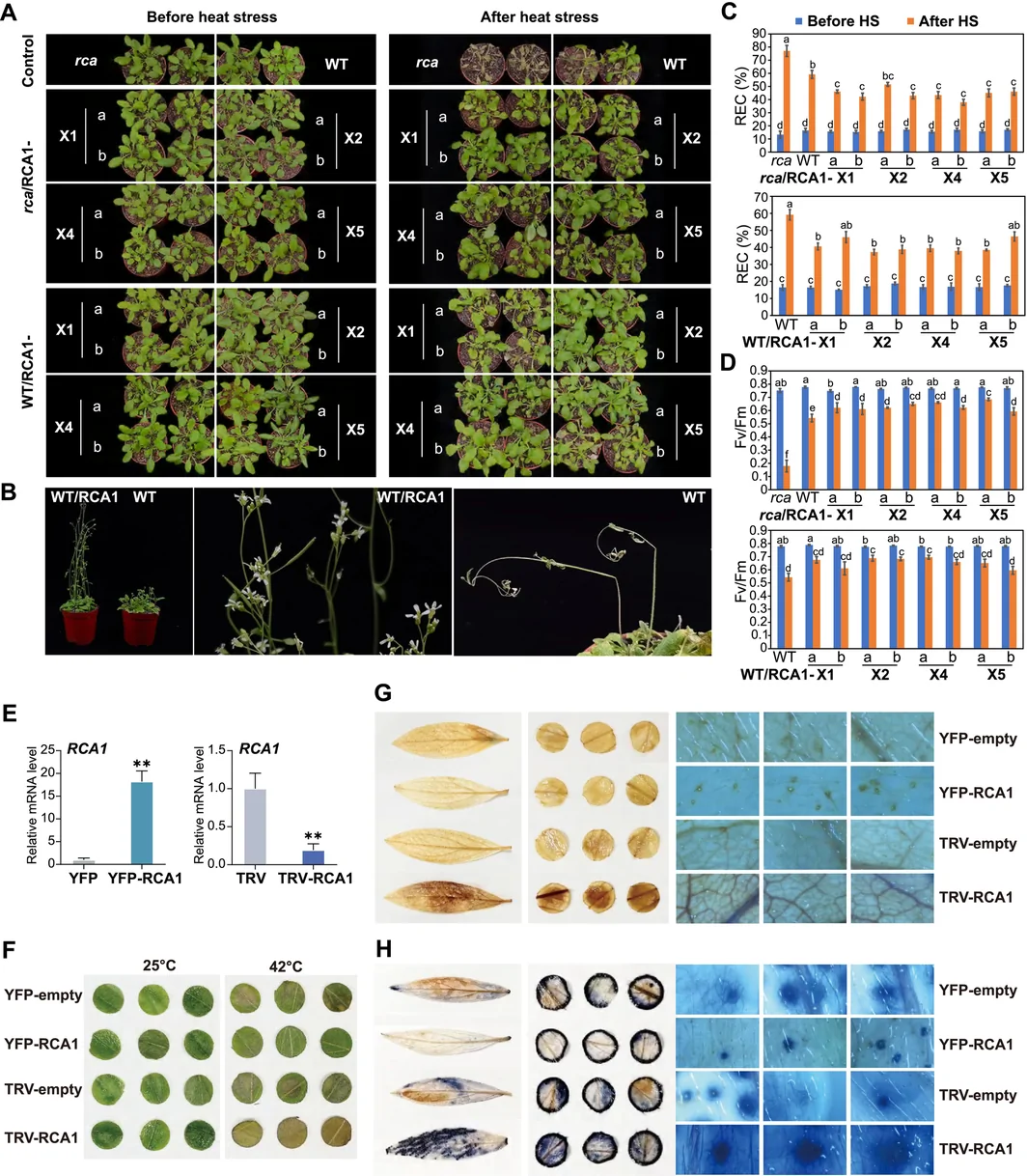
RCA1 alleviates heat‐induced injury in Arabidopsis and *R. hainanense* . (A) Phenotypes of WT, *rca*, *rca*/RCA1‐X1/2/4/5, and WT/RCA1‐X1/2/4/5 in Arabidopsis before and after heat stress (80 h at 45°C/40°C, day/night), following heat acclimation (37°C, 2 h) and recovery (25°C/20°C, 2 days). For each transcript variant, two independent homozygous lines (a, b), selected from the lines validated in Figures S3 and S4, were used. (B) Inflorescence phenotypes after heat stress. Left: bolting in WT/RCA1 compared with WT. Middle: enlarged view of normal flowering and fruit set of WT/RCA1. Right: withered inflorescences of WT. (C) Relative electrical conductivity (REC) and (D) *F*_*v*_/*F*_*m*_ values across different genotypes. Top: complementation lines. Bottom: overexpression lines. Values with different letters are significantly different according to a one‐way ANOVA followed by Duncan's test (*p* < 0.05). (E) Quantitative RT‐PCR analysis of *RCA1* expression in *R. hainanense* after transient overexpression or silencing. Statistical significance was determined by Student's *t*‐test (**p < 0.01). (F) Leaf disc phenotypes of *R. hainanense* subjected to transient *RCA1* overexpression (YFP‐RCA1) or silencing (TRV‐RCA1) and their corresponding vector controls before and after heat treatment (42°C, 12 h). (G) DAB staining for H_2_O_2_ accumulation, and (H) NBT staining for O^2−^ detection after heat stress. Left: whole‐leaf staining; middle: leaf disc staining; right: microscopic view of stained leaves at the same magnification. Data are the means ± SD (*n* = 3 for C, D and E).

Transmission electron microscopy revealed that heat stress severely disrupted chloroplast ultrastructure, particularly in the *rca* mutant, which exhibited swollen chloroplasts, disorganized thylakoids, and prominently enlarged osmiophilic granules (Figure S6). While wild‐type Arabidopsis also showed significant damage, both *RCA1*‐complemented and overexpressing lines maintained largely normal chloroplast morphology with only minor granule enlargement, demonstrating a protective role of RCA1 in chloroplast integrity under thermal stress. These results demonstrate that RCA1 mitigates heat‐induced photosynthetic injury, and the functional effects of its splice isoforms point to underlying redundancy.

For the homologous functional characterization of *RCA1* in *R. hainanense* , we exclusively used the X1 splice variant, which showed both superior thermoinduction and thermostability. Transient overexpression of *RCA1* significantly alleviated heat‐induced leaf damage, whereas its transient silencing markedly exacerbated the injury (Figure 2E,F). Further mechanistic insights were provided by histochemical staining: trypan blue (cell viability, Figure S7), DAB (H_2_O_2_) and NBT (O^2−^) staining revealed that overexpression lines accumulated fewer dead cells and lower levels of reactive oxygen species, whereas silenced tissues exhibited the opposite pattern (Figure 2G,H). Collectively, these results demonstrate that RCA1 plays a critical role in thermotolerance of *R. hainanense* .

### 2.3 Heat‐Induction of *RCA1* Is Mediated by HSFA2

Given that *RCA1* is strongly induced by heat stress (Figure 1), its promoter region was analysed to determine whether this heat responsiveness is conferred by transcriptional regulation and to identify the upstream factors involved. A 1622‐bp upstream fragment of *RCA1* was fused to a *GUS* reporter and transformed into Arabidopsis. This construct exhibited basal GUS activity at 25°C, but showed enhanced induction in green tissues following heat treatment at 37°C (Figure S8A). In dual‐luciferase reporter (DLR) assays, all three truncated promoter constructs drove heat‐induced expression in *Nicotiana benthamiana* (Figure S8B,C), suggesting that multiple cis‐regulatory elements are distributed across the promoter.

To identify the transcription factors responsible for this regulation, weighted gene co‐expression network analysis (WGCNA) was performed and revealed a strong association between *RCA1* and *HSFA2* (Figure S9), a central regulator of thermotolerance. Functional validation showed that HSFA2 exhibited transcriptional activation activity (Figure 3A), significantly enhanced *RCA1* promoter‐driven luciferase expression in DLR assays (Figures 3B and S10A), and increased *RCA1* transcript abundance upon transient overexpression in *R. hainanense* leaves (Figure 3C). DNA affinity purification sequencing (DAP‐seq) analysis of HSFA2 identified the canonical HSE as the most significantly enriched motif (nGAAnnTTCn, *p* = 1 × 10^−178^; Figure 3D). Furthermore, independent MACS2 peak calling for each replicate against the Input control identified HSFA2 enrichment within the *RCA1* promoter (Figure 3E). Electrophoretic mobility shift assay (EMSA) verification of four putative *cis*‐elements (E1–E4) demonstrated that HSFA2 specifically binds to E1 (GAACAATCTGGAA, an overlapping palindrome module) and E4 (GGGAGTTTC), both of which differ from the canonical HSE by a single nucleotide (Figure 3F). While two nucleotide mutations impaired HSFA2 binding to E1 but not to E4 (Figure 3G,H), three nucleotide mutations were needed to disrupt the binding to E4 (Figure 3I,J). Structural predictions using AlphaFold3 corroborated these findings, showing that HSFA2 formed more hydrogen bonds and exhibited higher binding confidence with the wild‐type E1 and E4 sequences than with their respective mutants (Figure S10B,C). Yeast one‐hybrid (Y1H) assays also confirmed binding of HSFA2 to E1 and E4 (Figure 3K). Collectively, these findings establish that HSFA2 directly binds atypical HSEs within the *RCA1* promoter and transcriptionally activates its expression, thereby linking heat stress signalling to *RCA1* induction.

**FIGURE 3:**
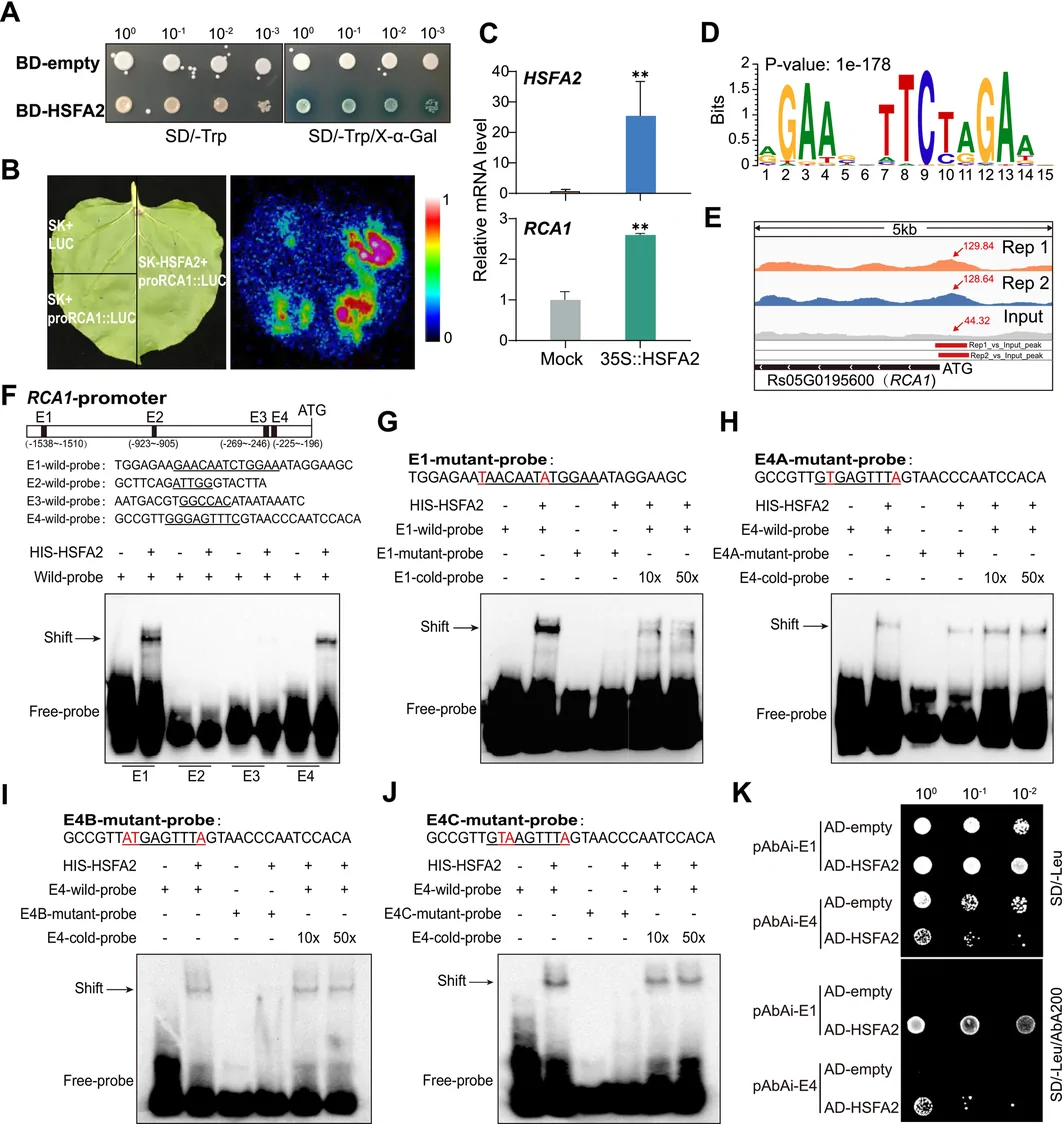
HSFA2 directly binds and transcriptionally activates the *RCA1* promoter under heat stress. (A) Transactivation activity of HSFA2 determined by yeast assays. (B) DLR assay showing activation of the *RCA1* promoter by HSFA2. Representative luminescence image is shown. (C) *RCA1* transcript levels in *R. hainanense* leaves after transient overexpression of *HSFA2*, quantified by qRT‐PCR. Statistical significance was determined by Student's *t*‐test relative to the mock control (**p < 0.01). (D) De novo motif analysis of HSFA2 DAP‐seq peaks, with the top enriched motif (nGAAnnTTCn, *p* = 1 × 10^−178^). (E) Genome‐browser view of HSFA2 DAP‐seq signals across the *RCA1* promoter. Peaks were independently called for each replicate (Rep1 and Rep2) against Input control and are displayed as Rep1_vs_Input_peak and Rep2_vs_Input_peak tracks; local signal maxima are annotated for visual comparison. (F) EMSA confirming HSFA2 binding to two atypical promoter elements, E1 and E4. ‘+’ and ‘−’ denote the presence/absence of recombinant protein (Figure S10D,E). (G) Competitive EMSA with biotinylated E1 probe (E1‐wild‐probe), unlabelled cold probe (E1‐cold‐probe), or mutant probe (E1‐mutant‐probe). 6 × HIS‐HSFA2: 8 μg recombinant protein. (H–J) EMSA validation of E4 element specificity using three mutant probes (E4A–C). (K) Y1H assays verifying HSFA2 binding to the E1 and E4 elements. Transformants were grown on SD/−Leu medium supplemented with 200 ng mL^−1^ AbA. Data are the means ± SD (*n* = 3 for C).

### 2.4 Temperature‐Dependent Co‐Activation of *RCA1* by the HSFA1–HSFA2 Complex

As a central regulator of thermotolerance, HSFA1 was investigated for its role in regulating *RCA1* expression. Under heat stress, *HSFA1* transcript levels showed a slight initial decrease followed by recovery, while its protein abundance accumulated to some extent (Figure 4A, top panels). In contrast, HSFA2 displayed an early peak and subsequent fall back at both transcript and protein levels (Figure 4A, bottom panels). Given the protein‐level accumulation of HSFA1, we further explored its regulatory effect on *RCA1*. Both EMSA and Y1H assays confirmed that HSFA1 specifically binds to the *cis*‐element E1 but not E4 in the *RCA1* promoter (Figures 4B,C and S11A). Structural predictions generated with AlphaFold3 supported these findings, revealing that HSFA1 formed more hydrogen bonds and exhibited higher binding confidence with the wild‐type E1 sequence compared to its mutant form (Figure S11B,C). Additionally, transient overexpression in *R. hainanense* and DLR assays demonstrated that HSFA1 functions as a transcriptional activator of *RCA1* (Figures 4D,E and S11D,E).

**FIGURE 4:**
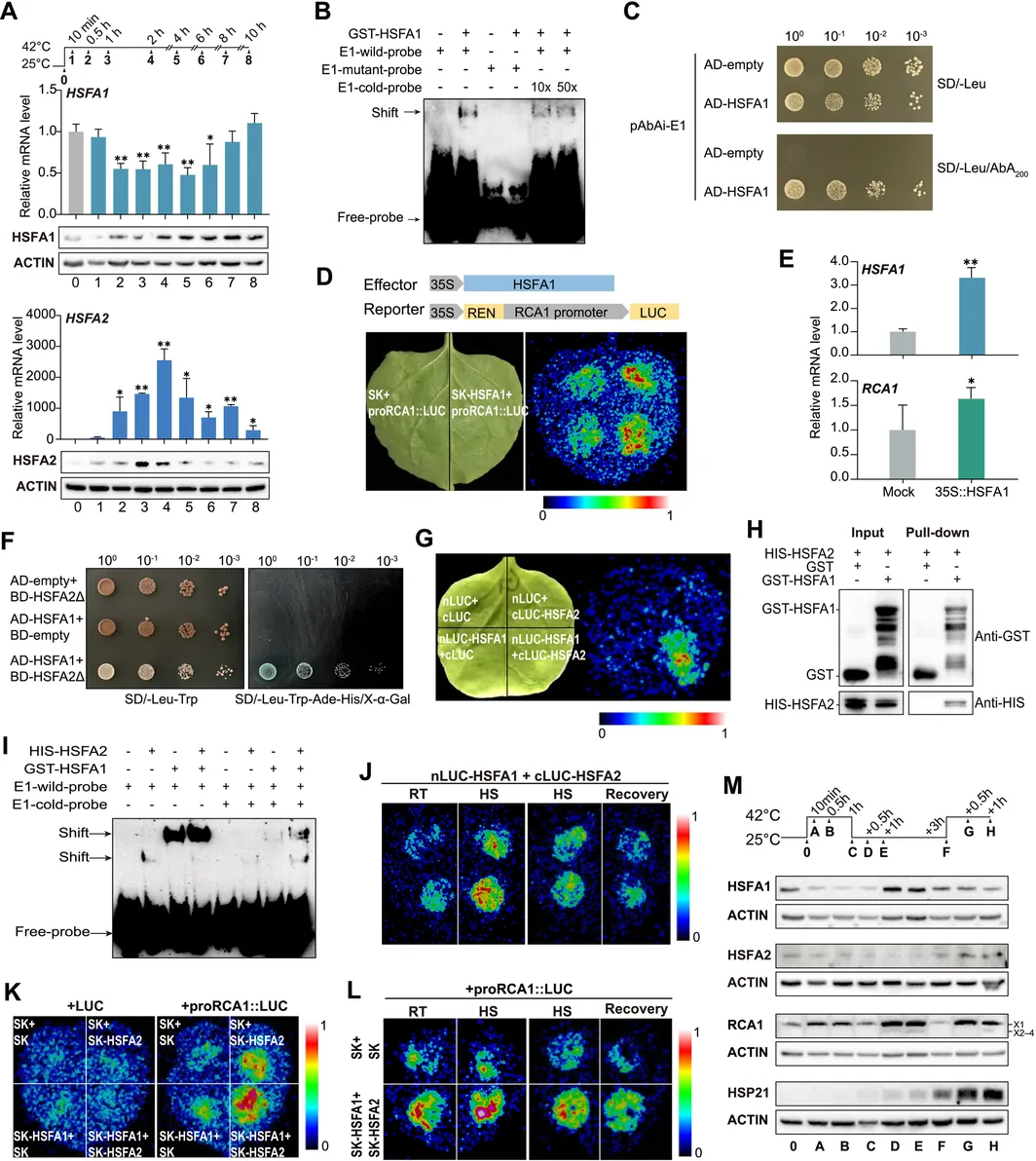
HSFA1 transcriptionally activates *RCA1* and forms a functional co‐activation complex with HSFA2 under heat stress. (A) Transcript (qRT‐PCR) and protein (Immunoblot) levels of HSFA1 and HSFA2 in *R. hainanense* under prolonged heat stress (42°C). The numbers (0–8) at the top of the panels indicate the duration of the heat treatment (in hours). Statistical significance was determined by Student's *t*‐test (*p < 0.05, **p < 0.01). (B) Binding specificity of HSFA1 to the E1 *cis*‐element. Recombinant GST‐HSFA1 (8 μg) (Figure S10F) was incubated with biotin‐labelled E1‐wild‐probe (10 nM). Specific binding was confirmed by competition with unlabelled E1‐cold‐probe and absence of binding to biotin‐labelled E1‐mutant‐probe. (C) Y1H assay confirming HSFA1 binding to the E1 in yeast cells. (D) DLR assay in *N. benthamiana* leaves showing HSFA1‐mediated activation of the pro*RCA1*::LUC construct. (E) Quantitative RT‐PCR analysis of *R. hainanense* leaves transiently overexpressing *HSFA1*, showing significant upregulation of *RCA1* transcript levels compared to the mock control. Statistical significance was determined by Student's *t*‐test relative to the mock control (*p < 0.05, **p < 0.01). (F) Y2H assay showing interaction between HSFA1 and the activation domain‐truncated HSFA2 (HSFA2ΔAHA). (G) LCI assay confirming HSFA1–HSFA2 interaction in living *N. benthamiana* leaves. (H) Pull‐down assay confirming the interaction between HSFA1 and HSFA2. Recombinant GST‐HSFA1 or GST control was incubated with HIS‐HSFA2, followed by immunoblotting with anti‐GST and anti‐HIS antibodies. The minor lower‐molecular‐weight bands observed in the GST‐HSFA1 immunoblot are typical trace‐level degradation products detected by the highly sensitive polyclonal antibody following the prolonged in vitro pull‐down incubation; the input protein showed high integrity on Coomassie‐stained gels (Figure S10D–F). (I) EMSA showing enhanced binding affinity to the E1 when HSFA1 and HSFA2 proteins were incubated together. (J) Synergistic activation of pro*RCA1*::LUC activity by co‐expression of HSFA1 and HSFA2 in *N. benthamiana* leaves. (K) Quantification of HSFA1‐HSFA2 interaction strength via LCI under room temperature, heat stress, and recovery conditions. (L) Dynamic activation of the *RCA1* promoter by the HSFA1‐HSFA2 complex under room temperature, heat stress, and recovery conditions, as measured by DLR assay. (M) Immunoblot analysis of HSFA1, HSFA2, RCA1, and HSP21 protein accumulation during temperature fluctuations (heat stress, recovery, second heat stress). A major band consistent with RCA1‐X1 and lower‐molecular‐weight signals within the predicted RCA1‐X2–X4 range is indicated. Data are the means ± SD (*n* = 3 for A and E).

Given that both HSFA1 and HSFA2 bind to the *RCA1* promoter, we investigated their potential interaction. Since HSFA2 exhibits intrinsic auto‐activation activity (Figure 3A), we deleted its AHA activation domain in the BD‐HSFA2 construct based on sequence alignment with *Solanum lycopersicum* HSFA2 (Figure S12A) (Döring et al. 2000; Chan‐Schaminet et al. 2009). The truncated construct no longer exhibited auto‐activation (Figure S12B), thereby ensuring the reliability of subsequent assays. Yeast two‐hybrid (Y2H), luciferase complementation (LCI), and pull‐down assays collectively confirmed that HSFA1 and HSFA2 physically interact, with their heteromeric association being stronger than the homomeric interaction of HSFA1 (Figures 4F–H and S13A). Notably, when co‐expressed, the HSFA1‐HSFA2 heterocomplex exhibited enhanced binding affinity to the *RCA1* promoter (Figure 4I) and stronger transcriptional activation (Figures 4J and S13B), indicating that the two factors act synergistically to co‐activate *RCA1* expression under heat stress. Structural modelling further indicated that HSFA1 and HSFA2 preferentially assemble into stable heterohexamers via their oligomerization domains, which then bind the promoter through their DNA‐binding domains (Figure S13C,D).

To simulate natural temperature fluctuations, we further examined this regulatory module under heat stress and recovery conditions. The HSFA1‐HSFA2 interaction, as well as their co‐activation of *RCA1*, strengthened during heat stress and diminished during recovery (Figures 4K,L and S13E,F). Under changing temperatures, the proteins exhibited delayed expression dynamics: the accumulation pattern of HSP21 resembled that of HSFA2, whereas RCA1 expression appeared to be co‐regulated by both HSFA1 and HSFA2 (Figure 4M). Notably, the immunoblot detected a major ~46 kDa RCA1 band consistent with the predicted molecular mass of RCA1‐X1, together with lower‐abundance signals in the 42–43 kDa range consistent with RCA1‐X2/X3/X4. Strikingly, upon a second heat shock, RCA1 protein accumulation increased sharply within 0.5 h, supporting a role of RCA1 in heat stress memory. Our results suggest that the pre‐formed HSFA1–HSFA2 complex poised at the promoter serves as a molecular switch, permitting rapid transcriptional activation under recurrent heat stress.

### 2.5 RCA1 Interacts With Multiple Chloroplast‐Localized Proteins

Given its rapid induction and high expression under heat stress, characteristics shared with HSPs, we hypothesized that RCA1 may function as a molecular chaperone contributing to thermotolerance. To identify its potential interacting partners, we employed a stepwise screening strategy. First, yeast library screening, combined with STRING‐based protein–protein interaction prediction and subcellular localization analysis, yielded 15 candidate interactors of RCA1‐X1 (all subsequent RCA1 protein experiments used this isoform). All of these were chloroplast‐localized and predominantly involved in photosynthesis‐related processes (full list in Table S1). These candidates were then validated using a combination of yeast two‐hybrid (Y2H), luciferase complementation imaging (LCI), bimolecular fluorescence complementation (BiFC), and GST pull‐down assays (Figures 5 and S14). Through this stepwise validation, four chloroplast‐localized proteins were confirmed as bona fide interactors of RCA1.

**FIGURE 5:**
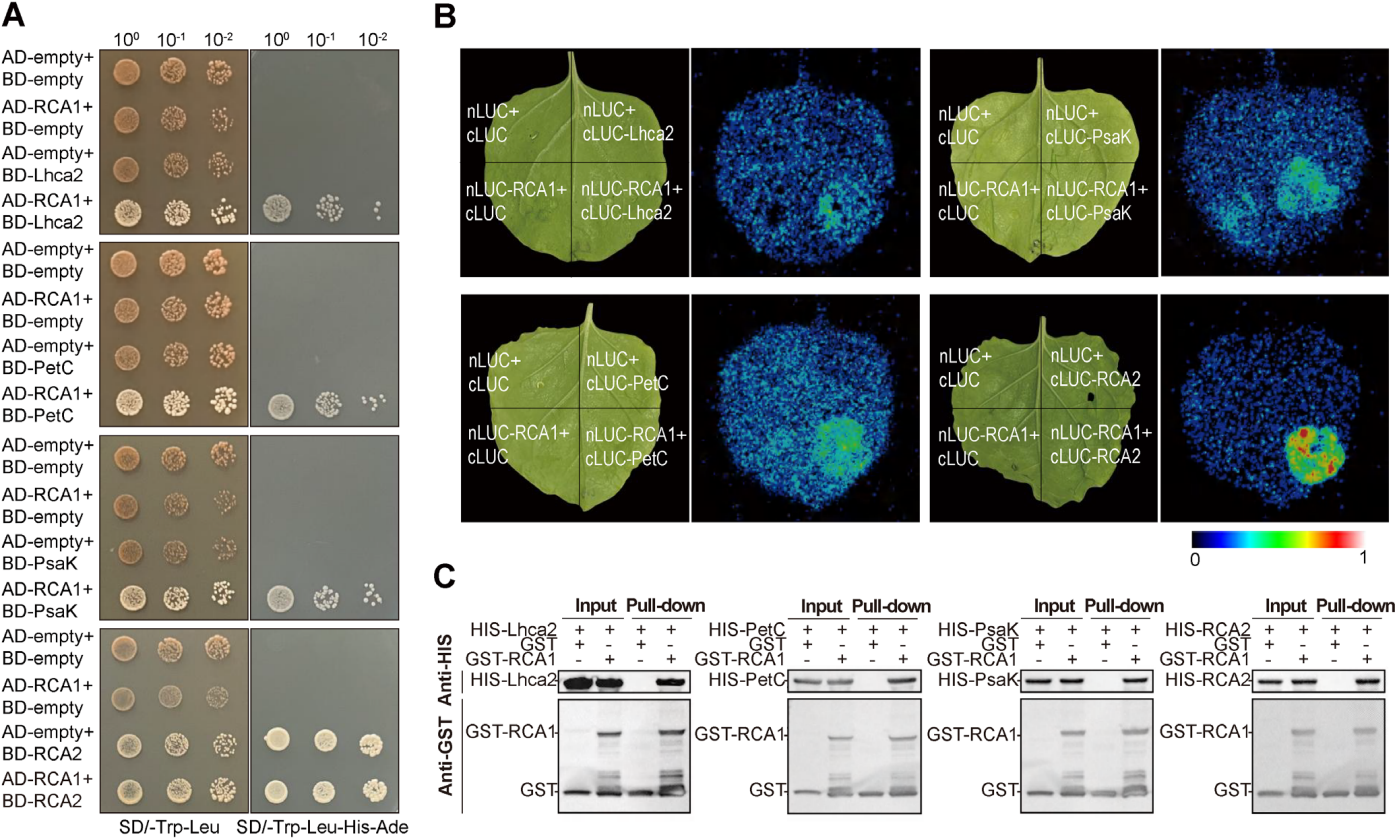
RCA1 interacts with photosynthetic complex subunits and its paralog RCA2. (A) Y2H assays showing interaction between RCA1 and Lhca2, PetC, or PsaK. Auto‐activation was observed for the RCA2 bait‐control combination. (B) LCI confirming the interaction between RCA1 and Lhca2, PetC, PsaK, or RCA2 in *N. benthamiana* leaves. (C) Pull‐down assays confirming the direct interactions between RCA1 and Lhca2, PetC, PsaK, or RCA2. For each target, lanes 1–2 are the input samples containing the 6 × HIS‐tagged target with GST or GST‐RCA1, respectively, whereas lanes 3–4 are the corresponding pull‐down fractions. Anti‐HIS detects the target proteins, and anti‐GST detects GST or GST‐RCA1. Enrichment of the 6 × HIS‐tagged target in the GST‐RCA1 pull‐down, but not the GST control, indicates direct interaction. Recombinant protein purification is shown in Figure S15.

Notably, these four interactors represent distinct subunits of photosynthetic complexes: Lhca2 (light‐harvesting complex), PetC (cytochrome b6f complex), PsaK (photosystem I), and RCA2 (an RCA member). In the Y2H system, RCA1 interacted with Lhca2, PetC, and PsaK. However, the RCA1‐RCA2 interaction could not be reliably assessed by Y2H due to auto‐activation when RCA2 was fused to the binding domain (Figure 5A). This technical limitation was overcome using *in planta* (LCI and BiFC; Figures 5B and S14C) and in vitro (GST pull‐down; Figure 5C) assays, which collectively confirmed all four interactions. The BiFC signals co‐localized with chlorophyll autofluorescence, indicating that these interactions occur within the chloroplast. Together, these assays demonstrate that RCA1 directly interacts with Lhca2, PetC, PsaK, and RCA2 in vitro and in planta.

### 2.6 RCA1 Exhibits Thermoprotective Activity Against Heat‐Induced Aggregation of Chloroplast Proteins

The conformational thermostability of RCA1 and its interacting chloroplast proteins was assessed using DSF. RCA1 demonstrated a *T*_*m*_ comparable to the molecular chaperone Cpn60β and significantly higher than those of Lhca2, PetC, PsaK, and RCA2 (Figure S16), indicating that RCA1 maintains intrinsic structural stability under thermal stress.

To evaluate whether this stability translates into a protective function beyond Rubisco activation, recombinant GST‐RCA1 was incubated with 6 × HIS‐tagged chloroplast proteins across a temperature gradient (25°C–75°C). RCA1 co‐incubation markedly increased the soluble‐fraction signal of all four partner proteins relative to the GST‐only control, indicating reduced heat‐triggered aggregation (Figure 6A). Optimal protection was observed at 45°C for RCA2 and Lhca2, and at 55°C for PetC and PsaK. This protective capacity was further examined in vivo using a heterologous yeast system. When co‐expressed with each chloroplast partner protein under heat stress at 37°C, RCA1 enhanced cellular thermotolerance, as evidenced by improved cell viability, increased optical density (OD_600_), and higher steady‐state accumulation of the target proteins compared with controls expressing the chloroplast proteins alone (Figure 6B–D).

**FIGURE 6:**
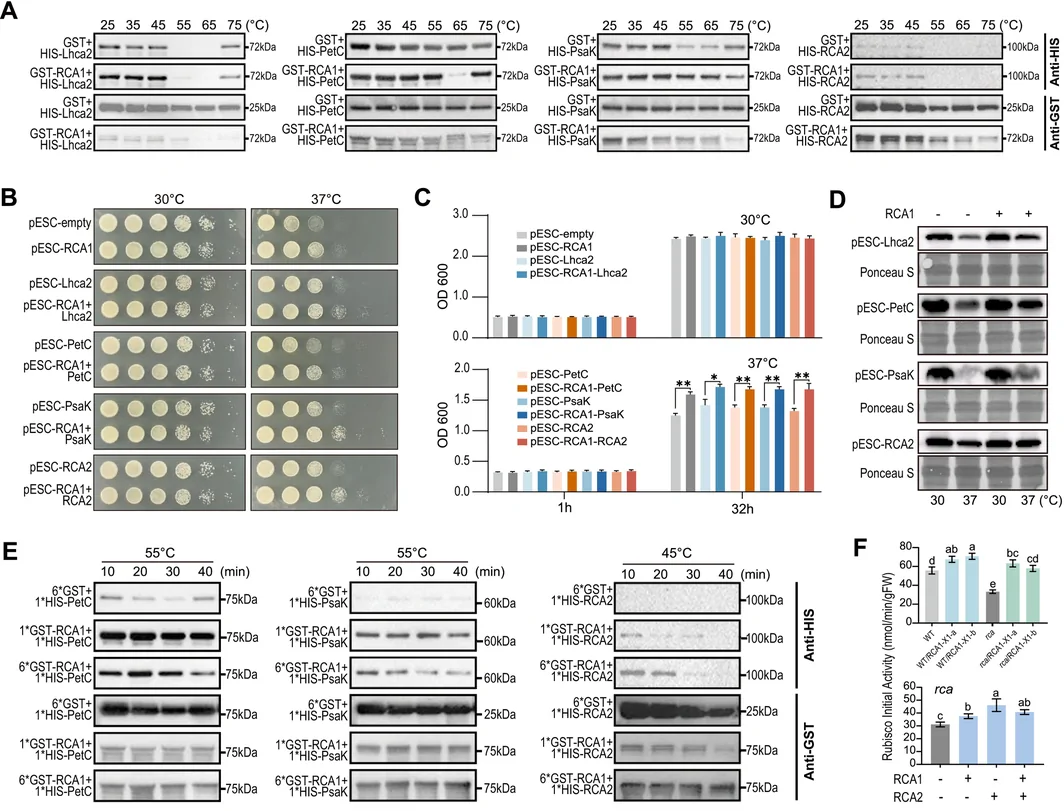
RCA1 exhibits thermoprotective activities distinct from its canonical Rubisco activation function. (A) In vitro thermoprotection assay. Soluble‐fraction immunoblot signals of 6 × HIS‐tagged chloroplast proteins (Lhca2, PetC, PsaK, RCA2) after co‐incubation with GST‐RCA1 across a temperature gradient (25°C–75°C). (B) Serial‐dilution growth assay of yeast expressing empty vector, RCA1 alone, each target protein alone, or RCA1 together with the indicated target protein under control (30°C) and heat‐stress (37°C) conditions. Cultures were adjusted to the same initial OD_600_ and subjected to 10‐fold serial dilutions before spotting. (C) Quantification of yeast growth in liquid culture under the same expression and temperature conditions as in (B). OD_600_ was measured at 1 h to verify comparable starting cell densities before temperature treatment and again at 32 h to assess subsequent growth. Statistical significance was determined by Student's *t*‐test (*p < 0.05, **p < 0.01). (D) Immunoblot analysis of target‐protein accumulation in yeast under control and heat‐stress conditions. Yeast cultures were harvested at the same cell density (OD_600_ = 1.0) before total‐protein extraction. Anti‐Myc immunoblotting detected the target proteins, and Ponceau S staining served as the loading control. (E) Effect of RCA1 stoichiometry (1× vs. 6× molar ratio of GST‐RCA1 to partner protein) on the soluble‐fraction signal of chloroplast proteins under heat treatment, using the assay described in (A). (F) Evaluation of Rubisco initial activity as an indicator of RCA1 efficacy. Top: Rubisco initial activity in Arabidopsis genotypes: *rca*, WT, *rca*/RCA1‐X1‐a/b, and WT/RCA1‐X1‐a/b. The suffixes ‘‐a’ and ‘‐b’ denote two independent homozygous transgenic lines for each genotype. Bottom: In vitro rescue of Rubisco initial activity in *rca* mutant extracts supplemented with recombinant RCA1, RCA2, or an equimolar mixture of both proteins. Values with different letters are significantly different according to a one‐way ANOVA followed by Duncan's test (*p* < 0.05). Data are the means ± SD (*n* = 3 for C and F).

Classical molecular chaperones, notably the small heat shock proteins, typically protect client proteins in a manner dependent on stoichiometry. The molar ratio between the chaperone and its client governs both the architecture of the resulting complexes and the efficiency of aggregation suppression (Yu et al. 2021). To determine whether the protective effects of RCA1 on its interacting proteins were similarly influenced by relative protein abundance, we compared the soluble retention of each partner protein at two molar ratios of GST‐fused RCA1 to partner protein (1× and 6×). Although RCA1 conferred significant thermoprotection on the partner proteins under both conditions, the response to elevated RCA1 abundance differed between RCA2 and the non‐RCA partners (Figure 6E). Protection of RCA2 was markedly enhanced at the 6× ratio, whereas PetC and PsaK were already efficiently protected at the equimolar 1× ratio. These results show that RCA1 directly suppresses the thermal aggregation of multiple chloroplast partner proteins through at least two distinguishable stoichiometric regimes. The protection of the non‐RCA partners supports a broader holdase‐like chaperone role beyond Rubisco activation, whereas the RCA1–RCA2 interaction is more consistent with heteromeric RCA complex assembly.

To assess the in vivo efficacy of RCA1 in maintaining the Rubisco activation state, we measured initial Rubisco carboxylation activity in different Arabidopsis genotypes (Figure 6F, top), revealing an activity hierarchy of: *rca < WT* < *rca*/RCA1‐X1 < WT/RCA1‐X1. To further dissect the relative contributions of RCA1 and RCA2 in restoring Rubisco activity, we performed an in vitro rescue assay by supplementing crude enzyme extracts from *rca* mutant leaves with the recombinant proteins and monitored bulk Rubisco carboxylation activity using a coupled‐enzyme assay. RCA2 alone elicited the strongest rescue of initial Rubisco activity; the RCA1 + RCA2 co‐treatment ranked second, and RCA1 alone produced a more modest restorative effect (Figure 6F, bottom). These observations indicate that, although RCA1 retains an intrinsic capacity to support Rubisco activation, RCA2 acts as the dominant RCA family member in sustaining carboxylation activity under the conditions tested. Conversely, the heat‐inducible RCA1, given its interactions with multiple photosynthetic subunits, may have evolved a specialized role in protecting the photosynthetic apparatus under thermal stress, a function extending beyond Rubisco activation.

### 2.7 Functional Divergence Among RCA Members in More Photosynthetic Organisms

Given the heat‐inducible and thermoprotective characteristics of RCA1, we hypothesized that RCA1 may have evolved as an adaptive response to high‐temperature environments. To investigate the evolution of *RCA* in other photosynthetic organisms, we collected 172 RCA protein sequences from 44 species (Table S2) and constructed a phylogenetic tree. The tree was divided into two clades: Clade I (containing RCA3) and Clade II (containing RCA1 and RCA2), comprising 50 and 122 sequences, respectively (Figure S17). Within Clade I but not Clade II, monocots and eudicots underwent independent evolutionary paths, with each experiencing internal duplication events (Figures S18 and S19). In eudicots, Clade II_E2 (containing RCA1) and Clade II_E3 (containing RCA2) originated from an early γ duplication event.

Nearly all RCA protein sequences contain the Rubisco activase domain (PLN00020) (Figure S20A). However, compared to Clade II, Clade I members exhibit differences in the N‐terminal domain and lack the C‐terminal CTE domain (Figure S20B). Within Clade II, only the β‐type RCAs in Solanaceae and Poaceae lack the CTE domain. Analysis of public expression data from several representative species across different tissues (Figure S21) and under abiotic stress (Figure S22) revealed consistently low expression of Clade I genes, while most Clade II genes showed high expression. These results suggest that Clade I may represent a contracting, functionally declining lineage, whereas Clade II is an expanding, functionally active lineage.

### 2.8 Convergent Evolution of Heat‐Inducible RCA in C4 Grasses of Poaceae and Woody Plants of *Rhododendron* Genus

Although heat‐inducible *RCA1* has been previously reported in grasses, we further analysed the Poaceae species within the monocot Clade II_M. These were divided into Pooideae and Panicoideae lineages, each containing α and β protein subclades (Figure 7A). By integrating phylogenetic relationships with chromosomal gene arrangement, we inferred that the grass ancestor possessed only a single copy of the *RCA* gene (Figure 7B). Following the divergence of major lineages, each underwent independent tandem duplication events, which led to subfunctionalization of the duplicated genes. During subsequent evolution, one *RCA* copy in rice lost its function due to multiple sequence deletions (Figure S23), resulting in its anomalous positioning in the phylogenetic tree (Figure 7A). In Pooideae, the α‐type is located downstream of the β‐type, whereas in Panicoideae, this order is reversed with the α‐type upstream (Figure 7B). In Panicoideae, besides considerable variation in the non‐conserved N‐ and C‐terminal domains, the α‐ and β‐types have six SNP sites that emerged in the central conserved domain, which may contribute to functional differentiation between α and β (Figure S24).

**FIGURE 7:**
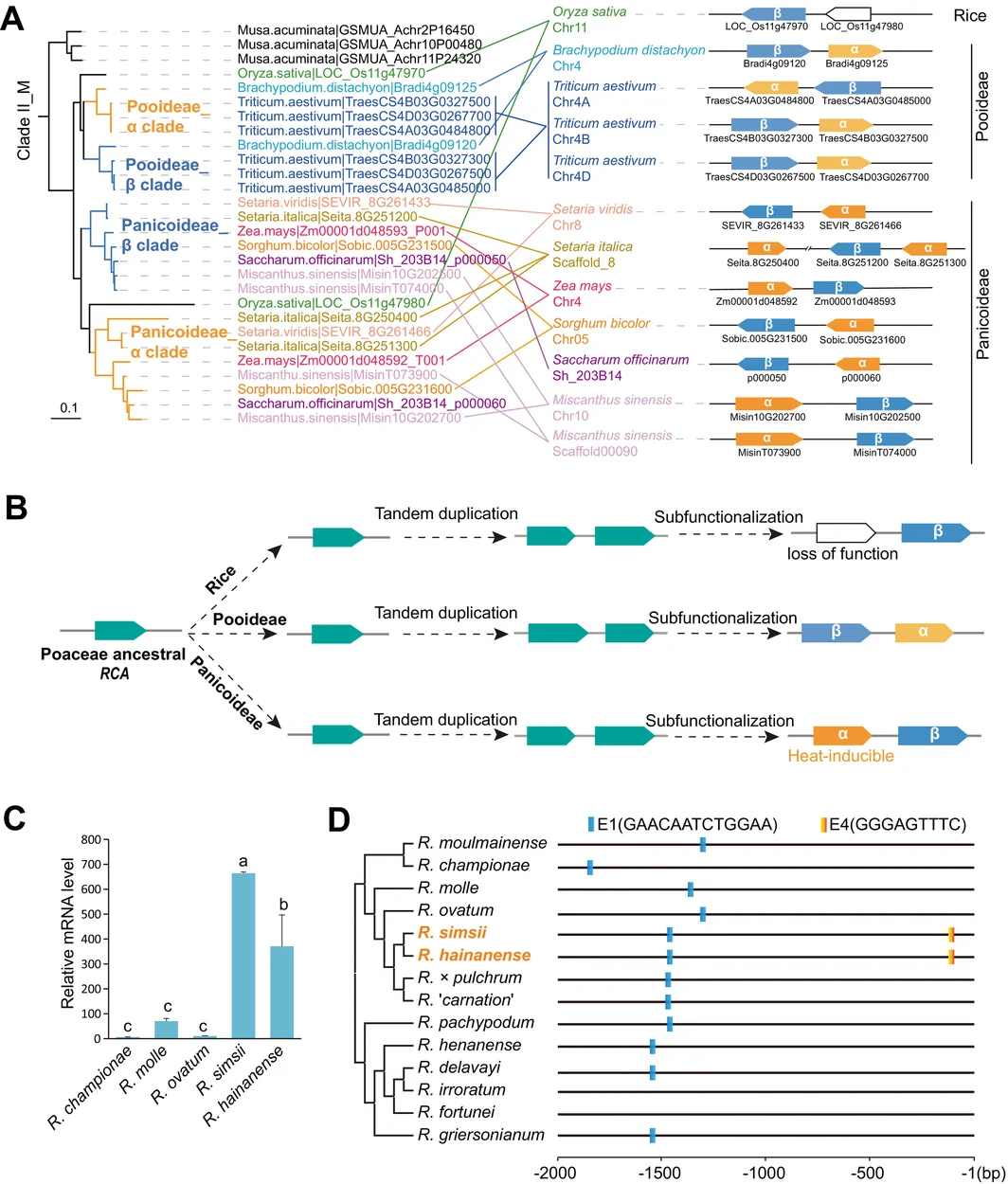
Phylogenetic evolution of *RCA* genes. (A) Phylogenetic tree and chromosomal arrangements of *RCA* genes in Poaceae. (B) Evolution of *RCA* in Poaceae. (C) Heat‐induced (37°C, 1 h) *RCA1* expression across *Rhododendron* species. Values with different letters are significantly different according to a one‐way ANOVA followed by Duncan's test (*p* < 0.05). Data are the means ± SD (*n* = 3). (D) Phylogenetic tree of *RCA1* sequences from 14 *Rhododendron* species and their corresponding promoter heat‐responsive elements.

Notably, we found that the Panicoideae (predominantly warm‐season C4) α‐type lineage evolved to acquire heat‐inducible properties. Previous studies have reported thermo‐inducible expression of α‐type RCAs in sorghum ( *Sorghum bicolor* ), maize, sugarcane ( *Saccharum officinarum* ), miscanthus ( *Miscanthus sinensis* ), and setaria ( *Setaria viridis* ) (Kim et al. 2021). Our expression analysis confirmed that the maize α‐type RCA (Zm00001d048592) is heat‐inducible, a feature absent in Arabidopsis, rice, and soybean ( *Glycine max* ) (Figure S22), highlighting a specific evolutionary adaptation to high‐temperature niches.

To assess whether the heat induction of *RCA1* is specific to *R. hainanense* , we first measured its heat‐induced expression across five *Rhododendron* species. The results showed differential *RCA1* expression patterns (Figure 7C). Notably, high levels of heat‐induced expression were detected not only in *R. hainanense* but also in the widely distributed *R. simsii*. Subsequently, we analysed the promoter regions of *RCA1* in more species (Figure 7D). While the E1 cis‐element was commonly present, the E4 element was identified exclusively in *R. hainanense* and *R. simsii*. Phylogenetic analysis placed these two species within the same clade. Interestingly, they are also the azaleas found at the lowest altitudes on Hainan Island. These findings collectively imply that the presence of the E4 element, a composite element bound by HSFA2 but not HSFA1, may be an evolutionary adaptation associated with heat tolerance in this particular lineage.

Together, these findings highlight a convergent evolutionary strategy where distantly related lineages, such as warm‐season grasses and woody perennials, have independently evolved specialized heat‐responsive *RCA* systems to survive in warming environments.

## 3 Discussion

The thermal sensitivity of RCA, a key regulator of Rubisco activation and photosynthetic carbon assimilation, is a critical kinetic bottleneck limiting photosynthetic efficiency under heat stress (Crafts‐Brandner and Salvucci 2000; Sage et al. 2008). Our work on the tropical perennial *R. hainanense* has uncovered a highly sophisticated, multi‐layered regulatory strategy centered on RCA1 to maintain photosynthetic integrity under heat stress (Figure 8). This strategy integrates rapid transcriptional synergy, dynamic alternative splicing, and a sophisticated protein‐level functional diversification that establishes RCA1 as a central adaptive hub for maintaining broad chloroplast proteostasis.

**FIGURE 8:**
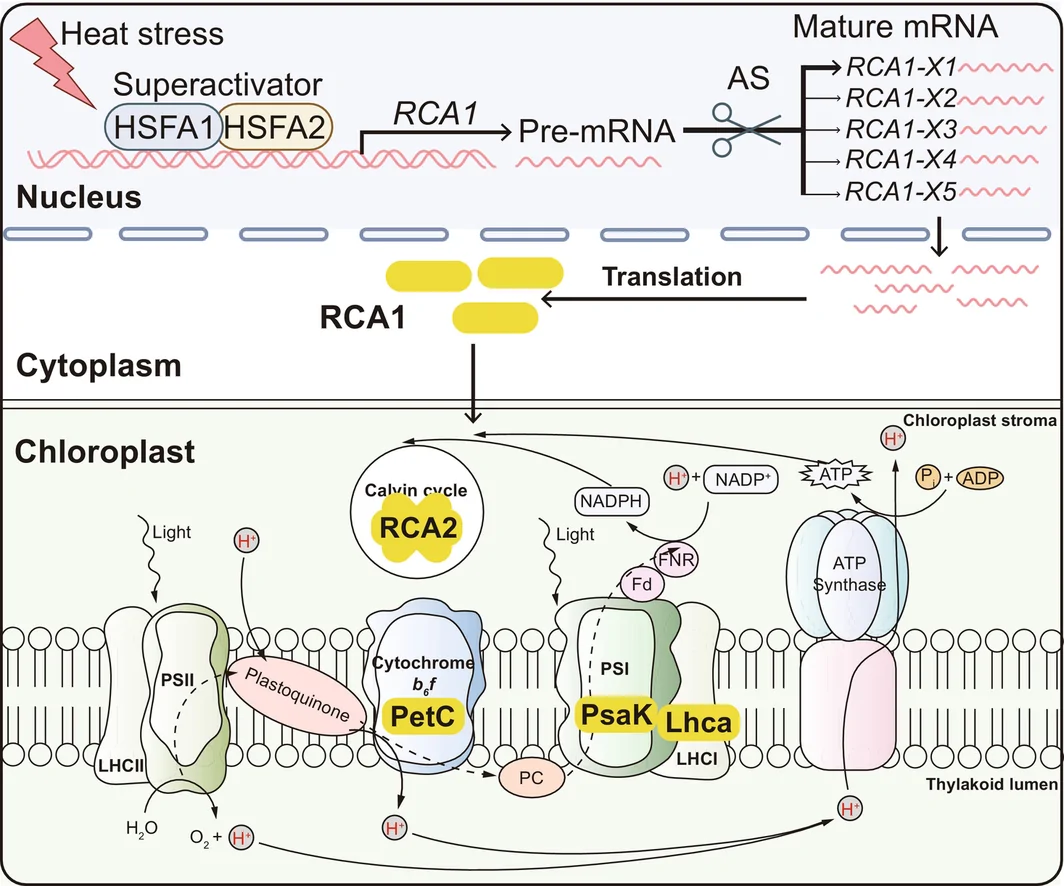
A schematic model of the multi‐layered regulatory strategy centred on RCA1 in *R. hainanense* under heat stress. The model integrates three layers of regulation centred on RCA1: transcriptional activation by the HSFA1–HSFA2 super‐activator complex, post‐transcriptional diversification via alternative splicing, and dual protein‐level functionality, in which RCA1 acts as a Rubisco activase that also assembles with RCA2 into heteromeric RCA complexes, and as a holdase‐like thermoprotective chaperone for non‐RCA chloroplast proteins such as Lhca2, PetC and PsaK, which together ensure photosystem protection under heat stress.

The rapid and robust induction of *RCA1* transcription is cooperatively regulated by the master regulator HSFA1 and the highly heat‐responsive HSFA2. In the broader context of RCA biology, while heat‐induced accumulation of *RCA* transcripts has been historically observed in species such as maize and cotton (Salvucci et al. 2003; Yin et al. 2014), the precise upstream transcriptional cascades governing this thermo‐responsiveness have remained largely elusive. While the HSFA1–HSFA2 hetero‐oligomeric complex has been reported to function as a super‐activator in tomato (Chan‐Schaminet et al. 2009), our findings reveal its specific recruitment for regulating a core photosynthetic component, RCA, thereby expanding the known regulatory scope of HSFs beyond classical heat shock proteins (Yoshida et al. 2011). Notably, this study demonstrates that HSFA2 acts as the primary determinant of *RCA1* induction, as it binds to both the E1 and the critical heat‐responsive E4 element within the *RCA1* promoter, whereas HSFA1 binds only to E1. In addition, we show that the activity of the HSFA1–HSFA2 complex is thermally modulated: it strengthens during heat stress and weakens upon recovery to normal temperatures. These dynamics support a model in which the promoter‐associated HSFA1–HSFA2 complex functions as a temperature‐responsive molecular switch, consistent with a role in transcriptional thermomemory (Pratx et al. 2025). These results suggest that HSFA1 may function as a co‐regulator that fine‐tunes HSFA2‐mediated activation and potentially assists in transcriptional resetting during recovery (Figure S25). This thermally gated, element‐specific cooperation likely enables *R. hainanense* to mount a rapid and reversible transcriptional response to fluctuating high temperatures in its native habitat. Together, the HSFA2–HSFA1–RCA1 module constitutes a key regulatory interface that directly links heat‐stress signalling to photosynthetic adjustment, allowing plants to dynamically optimize carbon assimilation under recurrent thermal stress.

Alternative pre‐mRNA splicing is an important regulator of plant heat‐stress responses, and AS regulation can also participate in heat‐priming establishment and HS memory maintenance (Ling et al. 2021). At the post‐transcriptional level, the single *RCA1* locus utilizes AS to generate five distinct transcript variants corresponding to four distinct protein isoforms. Within the broader RCA literature, the AS of a single *RCA* pre‐mRNA to generate α‐ and β‐isoforms is recognized as a conserved adaptive strategy in species such as Arabidopsis, spinach, and wheat (Werneke et al. 1989; Degen et al. 2020). However, the AS strategy in *R. hainanense* takes this mechanism to an unprecedented level of complexity. Different AS transcripts exhibit distinct expression profiles, with the α‐type X1 variant exhibiting superior ATPase activity and thermal stability (Figure 1), reflecting a clear evolutionary priority. While the exact physiological roles of the other variants (X2–X5) in vivo require further characterization, their differential expression patterns suggest distinct spatiotemporal regulation, potentially enabling functional complementarity. Together with the multiple immunoblot bands observed in azalea leaves (Figure 4M), which match the predicted molecular weights of the AS variants, these data indicate that the AS‐derived isoforms accumulate *in planta* and may constitute an integrated regulatory network that ensures the robustness of RCA1 function under heat stress.

A central advance of this work is the direct validation and mechanistic characterization of RCA1 as a dual‐function protein that combines its canonical AAA+ activase activity with holdase‐like protection of non‐RCA chloroplast proteins. Historically, the AAA+ chaperone activity of RCA has been regarded as primarily specific to Rubisco (Flecken et al. 2020; Portis 2003). Although Rokka et al. (2001) inferred that RCA might associate with thylakoid membranes under high temperatures, experimental validation of a generalized chaperone role had been lacking. We provide in vitro and heterologous evidence challenging the Rubisco‐exclusive paradigm, demonstrating that the heat‐inducible RCA1 exhibits a broader thermoprotective function beyond its canonical activase activity. In particular, RCA1 suppresses the thermal aggregation of the non‐RCA photosynthetic proteins, including the PSI‐associated subunits Lhca2 and PsaK and the cytochrome b6f complex subunit PetC (Figures 6 and 8), supporting a holdase‐like protective role analogous to that of classical molecular chaperones (Haslbeck and Vierling 2015; Waters and Vierling 2020). From a structural‐vulnerability perspective, the Rieske/PetC protein is a recognized chaperone‐dependent client even under non‐stress conditions, transiting through the chloroplast stroma where it is captured by Cpn60/Hsp70 prior to thylakoid insertion (Madueño et al. 1993); PsaK and Lhca2, as nuclear‐encoded thylakoid subunits, must likewise traverse the aqueous stroma as unassembled precursors before docking onto PSI. Heat stress further expands this stromal pool of vulnerable clients, both by accumulating newly imported precursors and by promoting partial disassembly of damaged photosystem supercomplexes (Aro et al. 2005). We propose that the heat‐inducible, thermostable RCA1, with a *T*_*m*_ comparable to Cpn60β (Figure S16), may act as a stromal holdase‐like factor that suppresses irreversible aggregation of these accessible precursor or unassembled protein states.

However, the RCA1‐RCA2 interaction is likely mechanistically distinct from those with the non‐RCA partners. RCA isoforms with different intrinsic thermal stabilities are known to form functional hetero‐oligomers. In the *Agave*/rice system, thermolabile rice RCA was selectively denatured upon heating, whereas thermostable *Agave* RCA remained functional (Shivhare and Mueller‐Cajar 2017). Similarly, in wheat, thermostable TaRca1‐β formed hetero‐oligomers with the more heat‐sensitive TaRca2, while elevated temperature resulted in loss of TaRca2 from the functional oligomeric pool and retention of TaRca1‐β activity (Scafaro et al. 2019). These observations indicate that heteromeric assembly does not abolish the intrinsic thermal sensitivity of the less stable RCA subunit. Our results address a complementary aspect of this process: RCA1 increased the amount of RCA2 retained in the soluble fraction after heat treatment, with greater RCA2 retention at a higher RCA1:RCA2 ratio. We therefore propose that RCA1 reduces the heat‐induced aggregation of soluble RCA2, potentially through RCA1‐RCA2 heteromeric assembly that favours retention of RCA2 in a soluble, assembly‐competent pool.

Furthermore, our findings offer novel insights into the potential role of RCA in heat‐acclimation memory. While the critical role of RCA in acute thermal response is well established, whether it actively participates in transcriptional thermomemory has remained an open question. Recent studies have established that HSFA2 is a master regulator of transcriptional heat memory (Lämke et al. 2016), and that pre‐mRNA alternative splicing functions as a critical layer of “splicing memory” during thermopriming (Ling et al. 2021). Our experimental observations place RCA1 at the intersection of these two mechanisms. When *R. hainanense* was subjected to a heat, recovery and reheat regime, RCA1 accumulated sharply within 0.5 h upon the second heat exposure, markedly faster than during the initial induction (Figure 4M), indicative of a primed state that enables rapid re‐induction of *RCA1* upon recurrent stress. Combined with the strict transcriptional dependence of *RCA1* on HSFA2 and its highly complex, stress‐responsive AS landscape, this rapid re‐induction strongly implies that RCA1 acts as a downstream effector of photosynthetic thermomemory. Although definitive proof will require long‐term, multi‐generational priming assays *in planta*, this HSFA1‐HSFA2‐RCA1 module likely primes the photosynthetic apparatus to endure subsequent, more severe thermal events.

Phylogenetic and expression analysis of *RCA* genes across photosynthetic organisms reveals a clear evolutionary and functional divergence, with Clade I likely representing a contracting lineage and Clade II exhibiting expansion and functional specialization. In Clade II, certain species have evolved specific adaptations in RCA to cope with thermal stress. Notably, the acquisition of critical promoter elements in *RCA1* of *R. hainanense* and *R. simsii*, which inhabit the hot, low‐altitude areas of Hainan Island, appears to be an adaptive trait that enables robust expression under heat stress and enhances thermotolerance. This gain of function represents a clear evolutionary neofunctionalization following gene duplication (Birchler and Yang 2022). The functional specialization of RCA1 (inducible, thermostable, thermoprotective chaperone) and RCA2 (constitutive, catalytic) represents a striking example of convergent evolution with the Panicoideae C4 grasses. These grasses, adapted to higher thermal optima, independently evolved dedicated, heat‐responsive α‐isoforms to sustain Rubisco activation (Kim et al. 2021), a strategy absent in many cool‐season Poaceae species (Perdomo et al. 2017, 2024). Similar to the pattern in *Rhododendron*, the divergence of grass α‐ and β‐isoforms following tandem duplication and subfunctionalization illustrates how gene duplication and functional differentiation can drive environmental adaptation. This recurring evolutionary trajectory, the decoupling of constitutive ‘housekeeping’ catalytic functions from inducible ‘emergency’ thermoprotective roles through gene duplication and specialization (Birchler and Yang 2022), provides a conceptual framework for engineering enhanced heat tolerance in crops.

In the pursuit of enhancing crop thermotolerance and photosynthetic efficiency under global warming, bioengineering RCA presents a direct and strategic approach (Long et al. 2025). While the introduction of complex carbon‐concentrating mechanisms (CCMs) into staple C3 crops like rice and wheat remains a formidable long‐term challenge (McGrath and Long 2014; Nguyen et al. 2024), engineering thermotolerant RCA offers a more immediate and tractable alternative. Previous efforts to engineer heat‐tolerant RCAs have followed two complementary directions: enhancing the intrinsic thermostability of constitutive RCA isoforms by site‐directed mutagenesis or by introducing warm‐adapted residues (Kurek et al. 2007; Kumar et al. 2009; Scafaro et al. 2019), and introducing or co‐expressing naturally thermostable RCA orthologs from heat‐adapted species, including thermostable RCA from the wild rice *Oryza australiensis* (Scafaro et al. 2016) and the co‐overexpression of Rubisco with RCA in rice (Suganami et al. 2021). Our work contributes a third, complementary route, exploiting a naturally evolved, heat‐inducible and thermostable RCA isoform that also exhibits broader thermoprotective activity toward chloroplast proteins. It is important to note that while heat‐inducible RCA isoforms exhibit superior thermostability, they often show lower catalytic activity under optimal conditions compared to their constitutive counterparts (Scafaro et al. 2016; Degen et al. 2020). To overcome this limitation, a promising strategy is to integrate the thermo‐stabilizing modules from heat‐inducible RCA with the high‐efficiency catalytic core of constitutive isoforms. Supported by machine learning‐guided protein design (Dilks et al. 2025), this rational engineering approach aims to create synthetic RCA variants that combine robust thermal resilience with high enzymatic activity, complementing rather than competing with previous Arabidopsis‐based engineering efforts. Such variants present a precise and feasible solution to sustain photosynthesis under heat stress, offering a more direct path to climate‐resilient crops than the systemic engineering required for full CCM implementation.

In summary, RCA1 in *R. hainanense* combines canonical Rubisco activase activity with a broader thermoprotective function against heat‐induced protein aggregation, governed by a multilayered regulatory network at both transcriptional and post‐transcriptional levels. The integrated mechanism forms an economical evolutionary solution, maximizing photosynthetic efficiency and rapid stress protection from limited genetic resources. The insights from the *R. hainanense* regulatory system, particularly the potent HSFA1‐HSFA2 transcriptional switch and the thermoprotective capacity of RCA1, are thus critical targets for engineering enhanced thermal resilience and mitigating the projected yield losses in staple cereal crops facing rising global temperatures.

## 4 Materials and Methods

### 4.1 Plant Materials and Heat Treatment Protocols

The study used two‐year‐old *R. hainanense* cuttings, tissue culture seedlings of *R. hainanense* , *N. benthamiana*, Arabidopsis Col‐0, and the leaky *rca* T‐DNA insertion mutant (SALK_003204). Plants were grown at 25°C/18°C (day/night), with 65% relative humidity, a 12‐h photoperiod, and a photon flux density of 150 μmol photons m^−2^ s^−1^. To standardize circadian effects, all heat treatments were consistently initiated at 1 h after the onset of the light period (9:00 AM, Zeitgeber Time 1). For heat treatments, only the temperature was altered while other conditions remained unchanged. For light/dark comparative assays, plants were exposed to light (150 μmol photons m^−2^ s^−1^) or complete darkness for 1 h at the indicated temperatures. Specific treatment conditions are detailed in the corresponding figure legends.

### 4.2 Generation of Transgenic Arabidopsis Lines

The full‐length coding sequences of four *RCA1* alternative splicing variants (X1, X2, X4 and X5) were independently cloned into a plant binary expression vector under the control of the CaMV 35S promoter and carrying a Basta resistance cassette as the selection marker. The recombinant plasmids were introduced into *Agrobacterium tumefaciens* strain GV3101 and transformed into wild‐type Arabidopsis (Col‐0) for overexpression and into the leaky *rca* T‐DNA insertion mutant (SALK_003204) for complementation. The SALK_003204 allele carries a T‐DNA insertion in the 5′ untranslated region (163 bp upstream of the start codon) and retains partial basal RCA expression, allowing viability under standard conditions (Shan et al. 2011).

Transformation was performed using the floral‐dip method. Transgenic T1 plants were selected on MS medium containing 20 mg/L Basta, and genomic integration was verified by PCR. Positive T1 lines were further confirmed by qRT‐PCR and immunoblot analysis. Homozygous T3 lines were identified based on 3:1 Mendelian segregation of Basta resistance in T2 progeny. For each variant, the two homozygous lines with the highest RCA1 expression (designated as a/b) were selected for subsequent analyses.

### 4.3 Phylogenetic Analysis of RCAs

Full‐length coding sequences of *RCA1*, *RCA2* and *RCA3* were obtained from *R. hainanense* transcriptome data (PRJNA579430). Additional RCA homologues were retrieved from the National Center for Biotechnology Information (NCBI) database (http://www.ncbi.nlm.nih.gov/) or Phytozome (https://phytozome‐next.jgi.doe.gov/). Protein sequences were aligned using MAFFT (v7.467) (Katoh and Standley 2013) and a phylogenetic tree was constructed using the Maximum Likelihood (ML) method implemented in FastTree (Price et al. 2009). All trees were visualized and refined using the online Interactive Tree of Life (iTOL) (https://itol.embl.de/) (Letunic and Bork 2019).

### 4.4 Recombinant Protein Expression and Purification

The coding sequences of RCA1 variants, heat shock transcription factors (HSFA1 and HSFA2), and chloroplast‐localized interacting proteins (Lhca2, PetC, PsaK and RCA2) were cloned into expression vectors with an N‐terminal 6 × HIS tag or GST tag. Recombinant plasmids were transformed into *Escherichia coli* Rosetta (DE3), and protein expression was induced with IPTG at 16°C. After sonication and centrifugation, soluble proteins were purified by Ni‐NTA (for HIS‐tagged) or glutathione affinity chromatography (for GST‐tagged). Purified proteins were buffer‐exchanged: *RCA1* variants used for downstream DSF and ATPase activity assays were exchanged into the desalting buffer specified by Scafaro et al. (2019) (20 mM Tris–HCl pH 8.0, 0.2 mM EDTA, 7.5 mM MgCl_2_, 1 mM DTT and 50 mM KCl), and the other proteins into PBS. Protein purity was verified by SDS‐PAGE before use.

### 4.5 Isolation and Activity Analysis of the Promoter

Three fragments of the *R. hainanense* *RCA1* promoter (1622, 771 and 400 bp upstream of the start codon) were cloned into pGreenII0800‐LUC and pCAMBIA3301‐GUS vectors and transformed into *Agrobacterium tumefaciens* GV3101. For transient expression, proRCA1::LUC constructs were infiltrated into *N. benthamiana* leaves. After 72 h, leaves were subjected to 25°C or 42°C for 3 h, and LUC activity was detected using Beetle Luciferin as substrate. For stable transformation, proRCA1::GUS constructs were introduced into *Arabidopsis*, and homozygous T3 lines were obtained via antibiotic screening and PCR verification. GUS staining was performed on 7‐ and 30‐day‐old plants under heat stress, followed by destaining and observation under a stereomicroscope.

### 4.6 Subcellular Localization Analysis

The *RCA1* coding sequence (CDS, stop codon excluded) was cloned into the pHB‐YFP vector using the ClonExpress II One Step Cloning Kit (Vazyme). The recombinant construct was transformed into Arabidopsis protoplasts following a high‐efficiency transformation protocol (Wu et al. 2009). Transfected protoplasts were incubated under light conditions for 16 h before observation using a laser scanning confocal microscope to determine the subcellular localization of the YFP‐tagged RCA1 protein. All primers used for vector reconstruction are listed in Table S3.

### 4.7 Quantitative RT‐PCR Assay

Total RNA was extracted from *R. hainanense* using the RNAprep Pure Plant Plus Kit (TIANGEN). cDNA was synthesized with the RT Reagent Kit with gDNA Eraser (Takara). Quantitative PCR was performed using TB Green Premix Ex Taq II (Takara) on a Bio‐Rad CFX Connect Real‐Time System. The *18S rRNA* and *Actin2* genes served as internal controls for *R. hainanense* and Arabidopsis, respectively. Relative expression levels were calculated using the 2^−ΔΔCT^ method, with at least three biological replicates per experiment. Primer sequences are listed in Table S4.

### 4.8 Chlorophyll Fluorescence Measurement

Chlorophyll fluorescence was measured in Arabidopsis seedlings after 20 min of dark adaptation using an Imaging Pulse‐Amplitude Modulation (PAM) Fluorometer (Heinz–Walz) with the MAXI program. Initial fluorescence (*F*_*o*_) was recorded upon exposure to the measuring light, followed by application of a 0.8‐s saturating pulse (4000 μmol photons m^−2^ s^−1^) to determine maximum fluorescence (*F*_*m*_). The maximum quantum yield of PSII was calculated as *F*_*v*_/*F*_*m*_ = (*F*_*m*_ − *F*_*o*_)/*F*_*m*_, where *F*_*v*_ represents variable fluorescence.

### 4.9 Y1H Assay

*Cis*‐acting elements from the *RCA1* promoter were cloned into the pAbAi bait vector and transformed into Y1HGold yeast cells. The full‐length coding sequences of HSFA1 and HSFA2 were cloned into the AD prey vector and co‐transformed with the pAbAi‐element construct. Transformants were selected on SD/−Leu plates containing 200 ng mL^−1^ AbA and incubated at 30°C for 3–4 days. Positive colonies were verified by PCR. The Matchmaker Gold Yeast One‐Hybrid Library Screening System (Clontech) was used throughout.

### 4.10 Y2H Assay and Self‐Activation Test

The Y2H assay was performed using the Matchmaker Gold Yeast Two‐Hybrid System (Takara). A heat‐treated *R. hainanense* leaf cDNA library was constructed from RNA pooled across multiple heat treatments (37°C for 0, 0.5, 1 h; 42°C for 0.5 h). The full‐length *RCA1‐X1* coding sequence was cloned into pGBKT7 (bait), and the cDNA library into the pGADT7 (prey). Bait and prey constructs were co‐transformed into Y2HGold cells using the PEG/LiAc method. Transformants were selected on SD/−Leu/−Trp, then on high‐stringency SD/−Trp/−Leu/–His/−Ade medium supplemented with 200 ng mL^−1^ AbA where appropriate, and incubated at 30°C for 3–4 days. Due to strong self‐activation of full‐length HSFA2 (confirmed by blue colony formation on X‐α‐Gal medium), a truncated version lacking the AHA motif (HSFA2ΔAHA) was used for subsequent assays. Interactions were verified by colony growth on high‐stringency medium and blue colour development on X‐α‐Gal‐supplemented medium. Empty vector combinations served as negative controls.

### 4.11 DLR and LCI Assay

For DLR assays, coding sequences of *HSFA1* and *HSFA2* were cloned into pGreenII 62‐SK (SK) vector, and the *RCA1* promoter into pGreenII 0800‐LUC. For LCI, *HSFA1*, *HSFA2*, *RCA1*, and chloroplast proteins (*Lhca2*, *PetC*, *PsaK*, *RCA2*) were cloned into pCAMBIA1300‐nLUC and pCAMBIA1300‐cLUC. All constructs were transformed into *A. tumefaciens* GV3101 (pSoup), resuspended to OD_600_ = 1.0 in infiltration buffer (10 mM MES, 10 mM MgCl_2_, 150 μM acetosyringone, pH 5.6), and infiltrated into *N. benthamiana* leaves. After 36–48 h, leaves were maintained at 25°C or subjected to 42°C for 15 min, following the protocol described by Wu et al. (2024). For LCI, luminescence was detected using D‐luciferin and captured with a cooled CCD imaging system. For DLR, firefly and *Renilla* luciferase activities were measured using the Dual‐Luciferase Assay System with a GloMax 96 Microplate Luminometer (Promega).

### 4.12 BiFC Assay

*RCA1* and chloroplast proteins (*Lhca2*, *PetC*, *PsaK* and *RCA2*) were cloned into the pSPYNE‐35S (nYFP) and pSPYCE‐35S (cYFP) vectors and introduced into *A. tumefaciens* GV3101. Bacterial suspensions with a final OD_600_ of 1.0 carrying nYFP and cYFP constructs were mixed at 1:1 and co‐infiltrated into *N. benthamiana* leaves. After 36–48 h, YFP fluorescence was detected at 505–530 nm and chloroplast autofluorescence at 650–710 nm by confocal microscopy.

### 4.13 EMSA

GST‐HSFA1 and 6 × HIS‐HSFA2 fusion proteins were expressed in *E. coli* Rosetta cells following IPTG induction at 16°C and purified using GST or Ni‐NTA resins, with purity verified by SDS‐PAGE. Binding reactions were performed using a chemiluminescent EMSA kit (Beyotime) according to the manufacturer's protocol.

### 4.14 Transient Overexpression Assay in *R. hainanense* Leaves

SK‐HSFA1 and SK‐HSFA2 vectors were transformed into *A. tumefaciens* GV3101 (pSoup). Bacterial cultures were resuspended in osmotic buffer (10 mM MES, 10 mM MgCl_2_, 150 μM acetosyringone, pH 5.6) and incubated in the dark at 20°C for 5 h. Young leaves from *R. hainanense* cuttings were micro‐wounded and subjected to vacuum infiltration for 1 h. After washing, leaves were placed on agar plates in darkness at 20°C for 72 h before RNA extraction and qRT‐PCR analysis.

### 4.15 Immunoblot Analysis

Total proteins were extracted from *R. hainanense* leaves using a modified phenol method, separated by 10% SDS‐PAGE, and transferred to PVDF membranes. Membranes were blocked for 1 h at room temperature with 5% skim milk in TBST, then incubated overnight at 4°C with primary antibodies: anti‐HSFA1 (1:2000), anti‐HSFA2 (1:2000), a specific anti‐RCA1 (1:4000, against the unique QAPMDSGTHYAVM epitope), and anti‐HSP21 (1:4000). After washing, membranes were incubated with HRP‐conjugated secondary antibodies (1:10000) for 1 h. Protein bands were visualized using ECL substrate.

### 4.16 Pull‐Down Assay

Pull‐down assays were performed using Glutathione Magnetic Agarose Beads (MedChemExpress, HY‐K0234) according to the manufacturer's instructions. Purified GST, GST‐RCA1, or GST‐HSFA1 was incubated with the equilibrated beads at 4°C with gentle rotation to immobilize the GST‐tagged bait proteins. After magnetic separation, the beads were washed with pre‐chilled PBS to remove unbound proteins. The appropriate 6 × HIS‐tagged prey protein (HSFA2, Lhca2, PetC, PsaK or RCA2) was then added and incubated with the bait‐bound beads at 4°C with gentle rotation. An aliquot of the reaction mixture was retained as the input sample before magnetic separation. Following incubation, the beads were collected using a magnetic separation rack and washed repeatedly with pre‐chilled PBS to remove unbound proteins. Proteins remaining associated with the beads constituted the pull‐down fraction and were used to assess whether the 6 × HIS‐tagged prey was retained through interaction with the GST‐tagged bait. Bound proteins were eluted in SDS‐PAGE loading buffer by heating at 95°C for 5 min, separated by SDS‐PAGE, and detected by immunoblotting with anti‐GST (1:4000) and anti‐HIS (1:4000) antibodies to detect the GST‐tagged bait and 6 × HIS‐tagged prey proteins, respectively. GST alone served as the negative bait control in all assays.

### 4.17 DAP‐Seq and Data Analysis

Halo‐HSFA2 fusion protein was expressed using the TNT SP6 Coupled Wheat Germ Extract System and incubated with a genomic DNA library prepared from *R. hainanense* leaves. An aliquot of the genomic DNA library prior to affinity enrichment was retained and used as the Input control. After stringent washing, bound DNA was sequenced on an Illumina NovaSeq 6000 platform. Reads were aligned with BWA‐MEM. Peaks were independently called for Rep1 and Rep2 against the Input library using MACS2 (fold enrichment ≥ 2, *p* ≤ 0.01), and the resulting peak intervals were visualized in the genome‐browser view. *De novo* motif discovery was performed with HOMER.

### 4.18 Interaction Prediction by AlphaFold3

Composite confidence scores were calculated as Confidence_score = 0.8 × ipTM + 0.2 × pTM (Homma et al. 2023), where pTM is the predicted template modelling score and ipTM is the interface predicted template modelling score (Zhang and Skolnick 2004; Xu and Zhang 2010). Predicted Aligned Error (PAE) matrices were extracted to assess inter‐chain prediction reliability for the HSFA1 homotrimer, the HSFA2 homotrimer, and the DNA‐bound heterohexameric complex.

### 4.19 Protein Thermostability Analysis by DSF

Protein thermal stability was evaluated using DSF as described by Scafaro et al. (2019). Purified RCA1 isoforms (X1, X2, X4, X5) or chloroplast proteins (Lhca2, PetC, PsaK, RCA2) were exchanged into desalting buffer (20 mM Tris–HCl pH 8.0, 0.2 mM EDTA, 7.5 mM MgCl_2_, 1 mM DTT, 50 mM KCl). Each 20‐μL reaction contained 8 μM protein, SYPRO Orange dye (1:2000), and 0.2 mM ATP. Thermal denaturation was monitored from 20°C to 100°C at 1°C min^−1^ on a real‐time PCR system. Fluorescence was recorded at 490/610 nm, and *T*_*m*_ values were derived by fitting normalized fluorescence data to a Boltzmann sigmoidal equation. Eight technical replicates were performed per isoform.

### 4.20 Stoichiometry‐Dependent Thermoprotection Assay

Purified GST‐RCA1 or GST control was pre‐incubated with 6 × HIS‐tagged chloroplast proteins (Lhca2, PetC, PsaK or RCA2) at molar ratios of 1:1 (2 μM each) or 6:1 (12 μM RCA1 and 2 μM partner protein) in HEPES buffer at 4°C for 2–3 h, according to the chaperone–client titration strategy previously applied to the small heat shock protein HSP21 (Yu et al. 2021). Mixtures were equilibrated at 4°C for 2 h (1×) or 3 h (6×) with constant rotation, then exposed to defined heat treatments (25°C, 35°C, 45°C, 55°C, 65°C and 75°C, 20 min for the gradient assay; 45°C or 55°C for 10–40 min for the time‐course assay) in a thermal cycler. After centrifugation at 12 000× *g* for 30 min at 4°C, soluble fractions were resolved by SDS‐PAGE and immunoblotted with anti‐GST and anti‐HIS antibodies.

### 4.21 Measurement of Initial Rubisco Carboxylation Activity and In Vitro Rescue Assay

Rubisco initial carboxylation activity was determined using a commercial coupled‐enzyme plant Rubisco activity assay kit (Suzhou Grace Biotechnology). Leaf tissue (0.1 g) from Arabidopsis genotypes (*rca* mutant, WT, *RCA1* complementation and overexpression lines) was homogenized in ice‐cold extraction buffer, centrifuged at 12 000× *g* for 10 min at 4°C, and the supernatant was used for activity measurement. NADH oxidation was monitored at 340 nm for 10 min, and activity was calculated from the initial rate and normalized to fresh weight (nmol NADH min^−1^ g^−1^ FW). For in vitro rescue, purified recombinant RCA1, RCA2, or an equimolar mixture (2 μM each) was added to *rca* mutant leaf extracts, and activity was measured under the same conditions. All treatments included three biological replicates, each with two technical replicates.

### 4.22 Measurement of ATPase Activity

ATPase activity of 6 × HIS‐tagged RCA1 variants was measured using a continuous spectrophotometric coupled‐enzyme assay adapted from Milward (2018). The reaction mixture contained 100 mM Tricine (pH 8.0), 20 mM KCl, 5 mM MgCl_2_, 6% PEG‐3350, 2 mM PEP, 0.2 mM NADH, 2 mM ATP, and coupling enzymes (PK, 745 units mL^−1^; LDH, 906 units mL^−1^). The reaction was initiated by adding purified RCA1 proteins, and NADH oxidation was monitored at 340 nm. The turnover number *k*_*cat*_^ATP^ (s^−1^) was calculated from the rate of absorbance change using the NADH molar extinction coefficient (6.22 × 10^−3^ M^−1^ cm^−1^). All assays were performed with three independent replicates.

### 4.23 Yeast Thermotolerance Assay Under Heterologous Expression

Yeast thermotolerance assay was performed following the strategy used to evaluate plant heat shock proteins as molecular chaperones in yeast (Schirmer et al. 1994) and to assess plant gene function via growth and OD_600_ readouts under defined temperature regimes (Chen et al. 2023). Lhca2, PetC, PsaK, RCA2 and RCA1 coding sequences were cloned into pESC‐TRP. Transformants were induced in SG/−Trp containing 2% galactose, spotted onto SG/−Trp solid medium, and incubated at 30°C for 24 h, then transferred to 30°C or 37°C for 48 h. Growth was monitored by OD_600_ at 1 and 32 h under the corresponding temperature conditions, providing a quantitative assessment of yeast thermotolerance. Total yeast proteins were extracted and analysed by immunoblotting with anti‐Myc antibody. Ponceau S staining served as the loading control. Target‐protein abundance was compared between yeast expressing the target protein alone and yeast co‐expressing the target protein with RCA1 to assess whether RCA1 helped maintain target‐protein accumulation under heat stress. Three independent biological replicates were performed for each condition.

### 4.24 Statistical Analysis

Unless otherwise stated, biological experiments were performed with at least three independent biological replicates, and data are presented as the means ± SD. Statistical analyses were conducted using GraphPad Prism version 9.0. Normality and homogeneity of variances were assessed using Shapiro–Wilk and Levene's tests, respectively. Comparisons between two groups were performed using two‐tailed Student's *t*‐test; among three or more groups, one‐way ANOVA followed by Duncan's multiple range test was used. Differences were considered significant at *p* < 0.05 and highly significant at *p* < 0.01.

## Author Contributions

Xiuyun Wang and Yiping Xia conceived and supervised this study. Yuqing Cao, Xiaomian Zhang and Fangqian Qu performed the key experiments. Mengxuan Liu, Zheng Li and Qianyi Hu contributed to parts of the experimental work. Yuqing Cao and Xiaomian Zhang wrote the manuscript. Bing Liu, Xianbin Gu and Hong Zhou analysed parts of the data. Xiuyun Wang, Yiping Xia and Liangsheng Zhang reviewed and edited the manuscript. All authors read and approved the final manuscript.

## Funding

This work was supported by the National Natural Science Foundation of China (nos. 32171847, 32371937) and the Zhejiang Science and Technology Major Program on Agricultural New Variety Breeding (2021C02071‐2).

## Conflicts of Interest

The authors declare no conflicts of interest.

## Supporting information


**Figure S1:** Molecular characterization of the *RCA* gene family in *R. hainanense* .
**Figure S2:** Functional characterization of RCA1 and its splice variants.
**Figure S3:** Molecular validation of RCA1 overexpression lines.
**Figure S4:** Molecular validation of RCA1 complementation lines.
**Figure S5:** Analysis of relative chlorophyll content in transgenic plants under heat stress.
**Figure S6:** Transmission electron micrographs of chloroplast ultrastructure in Arabidopsis leaves before and after heat stress.
**Figure S7:** Trypan blue staining of cell viability in R. hainanense leaves after heat stress.
**Figure S8:** Heat‐induced activity of the RCA1 promoter.
**Figure S9:** Weighted gene co‐expression network analysis (WGCNA) of RCA1 under heat stress.
**Figure S10:** Analysis of HSFA2 binding and transcriptional activation of the RCA1 promoter.
**Figure S11:** Analysis of HSFA1 binding and transcriptional activation of the RCA1 promoter.
**Figure S12:** Identification of the AHA activation domain in HSFA2 and validation of its truncated construct.
**Figure S13:** Functional and structural analysis of HSFA1–HSFA2 hetero‐oligomerization.
**Figure S14:** Validation of additional interactions between RCA1 and chloroplast proteins.
**Figure S15:** Affinity purification of recombinant RhRCA1 and candidate interacting proteins.
**Figure S16:** Thermostability analysis of RCA1 and chloroplast proteins.
**Figure S17:** Phylogenetic tree of RCA genes across photosynthetic organisms.
**Figure S18:** Phylogenetic tree of RCA Clade II.
**Figure S19:** Phylogenetic tree of RCA Clade I.
**Figure S20:** Motif analysis of RCA proteins.
**Figure S21:** Tissue expression patterns of RCA genes in representative organisms.
**Figure S22:** Expression patterns of RCA genes in response to abiotic stresses in representative plants.
**Figure S23:** Multiple sequence alignment of rice α‐type RCA with other sequences.
**Figure S24:** Multiple sequence alignment of the α‐ and β‐type RCA members in Panicoideae.
**Figure S25:** The working model of temperature‐dependent transcriptional regulation of RCA1 by HSFA1 and HSFA2 in R. hainanense.
**Table S1:** Predicted potential interacting proteins of RCA1.
**Table S2:** The RCA sequences used in phylogenetic tree.
**Table S3:** Primers used for vector reconstruction.
**Table S4:** Quantitative RT‐PCR primers.

## Data Availability

The DAP‐seq data have been deposited in the SRA under BioProject accession PRJNA1475883.
