# Supplementary material for: A Heat‐Inducible Rubisco Activase Safeguards Photosynthesis via Multilevel Regulation and Dual Function
Source: Plant Biotechnol J. 2026 Oct 20:10.1111/pbi.70774. Online ahead of print. doi: 10.1111/pbi.70774 (PMC13683984; doi:10.1111/pbi.70774)
Supplement: Supplementary file 1 [file PBI-9999-0-s001.docx]

**Supplementary information**

**A heat-inducible Rubisco activase safeguards photosynthesis via multilevel regulation and dual function**

Yuqing Cao^1#^, Xiaomian Zhang^1#^, Fangqian Qu^1#^, Mengxuan Liu^1^, Zheng Li^1^, Bing Liu^2^, Xianbin Gu^3^, Qianyi Hu^1^, Hong Zhou^1^, Liangsheng Zhang^1^, Yiping Xia^1*^ and Xiuyun Wang^1*^

**The following Supporting Information is available for this article:**

Figure S1. Molecular characterization of the *RCA* gene family in *R. hainanense*.

Figure S2. Functional characterization of RCA1 and its splice variants.

Figure S3. Molecular validation of *RCA1* overexpression lines.

Figure S4. Molecular validation of *RCA1* complementation lines.

Figure S5. Analysis of relative chlorophyll content in transgenic plants under heat stress.

Figure S6. Transmission electron micrographs of chloroplast ultrastructure in Arabidopsis leaves before and after heat stress.

Figure S7. Trypan blue staining of cell viability in *R. hainanense* leaves after heat stress.

Figure S8. Heat-induced activity of the *RCA1* promoter.

Figure S9. Weighted gene co-expression network analysis (WGCNA) of *RCA1* under heat stress.

Figure S10. Analysis of HSFA2 binding and transcriptional activation of the *RCA1* promoter.

Figure S11. Analysis of HSFA1 binding and transcriptional activation of the *RCA1* promoter.

Figure S12. Identification of the AHA activation domain in HSFA2 and validation of its truncated construct.

Figure S13. Functional and structural analysis of HSFA1–HSFA2 hetero-oligomerization.

Figure S14. Validation of additional interactions between RCA1 and chloroplast proteins.

Figure S15. Affinity purification of recombinant RhRCA1 and candidate interacting proteins.

Figure S16. Thermostability analysis of RCA1 and chloroplast proteins.

Figure S17. Phylogenetic tree of *RCA* genes across photosynthetic organisms.

Figure S18. Phylogenetic tree of *RCA* Clade II.

Figure S19. Phylogenetic tree of *RCA* Clade I.

Figure S20. Motif analysis of RCA proteins.

Figure S21. Tissue expression patterns of *RCA* genes in representative organisms.

Figure S22. Expression patterns of *RCA* genes in response to abiotic stresses in representative plants.

Figure S23. Multiple sequence alignment of rice α-type RCA with other sequences.

Figure S24. Multiple sequence alignment of the α- and β-type RCA members in Panicoideae.

Figure S25. The working model of temperature-dependent transcriptional regulation of *RCA1* by HSFA1 and HSFA2 in *R. hainanense*.

Table S1. Predicted potential interacting proteins of RCA1.

Table S2. The *RCA* sequences used in phylogenetic tree.

Table S3. Primers used for vector reconstruction.

Table S4. Quantitative RT-PCR primers.

**Figure S1. Molecular characterization of the RCA gene family in *R. hainanense*.**

**(A)** Multiple sequence alignment of RCA1, RCA2, and RCA3 proteins. Conserved residues are shaded in black, highlighting regions of sequence identity, while divergent regions are unshaded.

**(B)** Phylogenetic analysis of RCA proteins from *R. hainanense* and seven other plant species, including model plants (Arabidopsis, rice) and close relatives (e.g., *R. simsii*, *Camellia sinensis*).

**(C)** FPKM values of *RCA1*, *RCA2* and *RCA3* in *R. hainanense* leaves after 1 h of heat treatment at 25 °C, 37 °C and 42 °C, derived from transcriptome profiling.

**(D)** Multiple sequence alignment of the five major *RCA1* splice variants (X1, X2, X3, X4, X5). The alignment is shown for the downstream region containing the sequence blocks that diverge among isoforms.

**(E)** Multiple sequence alignment of the five major RCA1 splice isoforms (X1, X2, X3, X4, X5). Distinct conserved domains are colored differently. Truncated regions resulting from alternative splicing are clearly delineated.

**Figure S2. Functional characterization of RCA1 and its splice variants.**

**(A)** Subcellular localization of RCA1-YFP fusion proteins in Arabidopsis mesophyll protoplasts. YFP signals (yellow) overlapped with chlorophyll autofluorescence (red), confirming chloroplast targeting.

**(B)** Expression levels of RCA1 variants under light and dark conditions. Leaves of *R. hainanense* were treated at standard temperature (25 °C) or heat stress (37 °C) for 1 h. The treatments were conducted under either standard continuous light conditions (photon flux density of 150 μmol photons m⁻² s⁻¹) or complete darkness.

**(C)** Differential scanning fluorimetry (DSF) analysis of RCA1 splice isoforms. Samples were heated at 1 °C min⁻¹, and fluorescence units (FU) normalized to the maximum value were recorded. Curves are the ordinary least-squares fit of a Boltzmann sigmoidal equation.

**(D–G)** Expression and purification of recombinant proteins. Affinity purification of RCA1 alternative splicing variants; D: RCA1-X1, E: RCA1-X2, F: RCA1-X4, G: RCA1-X5; M: 180 kDa protein marker; 2, 10, 18, and 26: filtrate; 1: column flow-through; 3, 11, 19, and 27: wash fraction; 4–9, 12–17, 20–25 and 28–33: imidazole gradient elution (each group: 10, 40, 60, 80, 100, 120 mM); red arrows indicate proteins to be further concentrated by ultrafiltration.

**Figure S3. Molecular validation of RCA1 overexpression lines.**

**(A)** Genomic DNA PCR analysis confirming successful T-DNA integration in transgenic overexpression lines (WT/RCA1-X1/2/4/5). M5: DL 5000 DNA Marker.

**(B)** Heatmap of *RCA1* mRNA abundance in overexpression lines. Relative expression levels were determined by qRT-PCR, normalized to the *Actin2* reference gene, and visualized as a color gradient. Based on the screening results, two homozygous lines with the highest *RhRCA1* relative expression were selected from each transcript variant for subsequent experiments. The selected lines were X1#3, X1#8, X2#4, X2#9, X4#6, X4#7, X5#2 and X5#9, hereafter referred to as WT/RCA1-X1-a/b, WT/RCA1-X2-a/b, WT/RCA1-X4-a/b and WT/RCA1-X5-a/b, respectively.

**Figure S4. Molecular validation of RCA1 complementation lines.**

**(A)** Quantitative RT-PCR analysis of *AtRCA* expression in the *rca* mutant of Arabidopsis. Data are the means ± SD (n = 3). Statistical significance was determined by Student's *t*-test (***P* < 0.01).

**(B)** Genomic DNA PCR analysis confirming successful T-DNA integration in transgenic complementation lines (*rca*/RCA1-X1/2/4/5). M5: DL 5000 DNA Marker.

**(C)** Heatmap of *RCA1* mRNA abundance in complementation lines. Relative expression levels were determined by qRT-PCR, normalized to the *Actin2* reference gene, and visualized as a color gradient. Based on the screening results, two homozygous lines with the highest *RhRCA1* relative expression were selected from each transcript variant for subsequent experiments. The selected lines were X1#10, X1#12, X2#8, X2#11, X4#1, X4#12, X5#8 and X5#14, hereafter referred to as *rca*/RCA1-X1-a/b, *rca*/RCA1-X2-a/b, *rca*/RCA1-X4-a/b and *rca*/RCA1-X5-a/b, respectively.

**Figure S5. Analysis of relative chlorophyll content in transgenic plants under heat stress.**

Measurement of relative chlorophyll content (SPAD value) in WT, *rca* mutant, and transgenic lines under heat stress. Data are the means ± SD (n = 3). Values with different letters are significantly different according to a one-way ANOVA followed by Duncan's test (*P* < 0.05).

**Figure S6. Transmission electron micrographs of chloroplast ultrastructure in Arabidopsis leaves before and after heat stress.**

Transmission electron micrographs of chloroplast ultrastructure in Arabidopsis leaves before and after heat stress. O: osmiophilic granules; S: starch grain; GL: grana lamellae; SL: stroma lamellae.

**Figure S7. Trypan blue staining of cell viability in *R. hainanense* leaves after heat stress.**

Trypan blue staining of cell viability after heat stress. Left: whole leaf staining; Middle: leaf disc staining; Right: micro view of stained leaves at the same magnification level. Data are the means ± SD (n = 3).

**Figure S8. Heat-induced activity of the RCA1 promoter.**

**(A)** Quantitative analysis and histochemical staining of GUS activity in pro*RCA1*::GUS transgenic Arabidopsis seedlings under room temperature (25 °C) and after 3 h of heat stress (37 °C). WT seedlings served as the control. Statistical significance was determined by Student's *t*-test (***P* < 0.01).

**(B)** Upper panel: Schematic diagram of truncated *RCA1* promoter fragments (P1–P3). Lower panel: Representative LUC signals in *N. benthamiana* leaves under 25 °C and 37 °C.

**(C)** Measurement of LUC/REN ratio in *N. benthamiana* leaves expressing three different *RCA1* promoter truncations (P1, P2, P3, as detailed in panel B) under room temperature (25 °C) and heat stress (42 °C) conditions. LUC/REN represents the ratio of firefly to *Renilla* luciferase activity. Data are the means ± SD (n = 3). Statistical significance was determined by Student's *t*-test (**P* < 0.05, ***P* < 0.01).

**Figure S9. Weighted gene co-expression network analysis (WGCNA) of RCA1 under heat stress.**

WGCNA of *RCA1* under heat stress. Nodes represent genes, with node size proportional to correlation strength.

**Figure S10. Analysis of HSFA2 binding and transcriptional activation of the RCA1 promoter.**

**(A)** Measurement of LUC/REN ratio in *N. benthamiana* leaves co-expressing the pro*RCA1*::LUC reporter and the SK-HSFA2 effector construct. LUC/REN represents the ratio of firefly to *Renilla* luciferase activity.

**(B)** Quantitative comparison of predicted binding confidence scores between HSFA2 and the wild-type versus mutant DNA probes, as calculated by AlphaFold3. Data are the means ± SD (n = 3). Statistical significance was determined by Student's *t*-test (**P* < 0.05, ***P* < 0.01, ****P* < 0.001). Ns, not significant.

**(C)** Molecular visualization of the predicted HSFA2-DNA binding interface. Protein structures are shown in blue, wild-type (left) and mutant (right) DNA elements in gold, and predicted hydrogen bonds as red dashed lines. DNA sequences are annotated below, with the JASPAR-predicted binding motif underlined and AlphaFold3-predicted nucleotide contact sites highlighted in blue.

**(D–G)** Expression and purification of recombinant proteins. Affinity purification of HIS-empty, HIS-HSFA2, GST-empty and GST-HSFA1. M: 180 kDa protein marker; 6, 13, 18: filtrate; 7, 14, 19: wash fraction; 15–17: GSH gradient elution (5, 10, 20 mM); 20–23: GSH gradient elution (0.5, 1, 3, 5 mM); 1–5 and 8–12: imidazole gradient elution (each group: 10, 40, 60, 80, 100 mM); red arrows indicate proteins to be further concentrated by ultrafiltration.

**Figure S11. Analysis of HSFA1 binding and transcriptional activation of the RCA1 promoter.**

**(A)** EMSA showing specific binding of recombinant HSFA1 to the biotin-labeled E1-wild-probe of the *RCA1* promoter, but not to the biotin-labeled E4-wild-probe.

**(B)** Quantitative comparison of predicted binding confidence scores between HSFA1 and the wild-type versus mutant DNA probes, as calculated by AlphaFold3.

**(C)** Molecular visualization of the predicted HSFA1-DNA binding interface. Protein structures are shown in blue, wild-type (top) and mutant (bottom) DNA elements in gold, and predicted hydrogen bonds as red dashed lines. DNA sequences are annotated below, with the JASPAR-predicted binding motif underlined and AlphaFold3-predicted nucleotide contact sites highlighted in blue.

**(D)** Measurement of LUC/REN ratio in *N. benthamiana* leaves co-expressing the pro*RCA1*::LUC reporter and the SK-HSFA1 effector construct.

**(E)** Yeast transcriptional activation assay: BD-HSFA1 fusion protein activated reporter gene expression, indicated by blue colonies on SD/-Leu-Trp/X-α-Gal medium.

LUC/REN represents the ratio of firefly to *Renilla* luciferase activity. Data are the means ± SD (n = 3). Statistical significance was determined by Student's *t*-test (**P* < 0.05, ****P* < 0.001).

**Figure S12. Identification of the AHA activation domain in HSFA2 and validation of its truncated construct.**

**(A)** Alignment of amino acid sequences of HSFA2 from *R. hainanense* and that from *S. lycopersicum*. The conserved DBD motif is shown in the yellow box, the OD motif in the orange box, the NLS motif in the green box, the AHA motif in the red box, and the NES motif in the purple box.

**(B)** Detection of HSFA2ΔAHA transcriptional activation activity using the yeast system. A yeast autoactivation assay was performed to verify whether the bait protein HSFA2ΔAHA exhibits intrinsic self-activation capacity.

**Figure S13. Functional and structural analysis of HSFA1–HSFA2 hetero-oligomerization.**

**(A)** Comparison of predicted binding confidence scores between the HSFA1–HSFA2 heterocomplex and the respective HSFA1 or HSFA2 homomers.

**(B)** Transcriptional synergy assay measuring LUC/REN ratio in *N. benthamiana* leaves co-expressing the pro*RCA1*::LUC reporter with both SK-HSFA1 and SK-HSFA2 effectors.

**(C)** Structural models of HSF complex assembly predicted by AlphaFold3, depicting homomeric complexes (up) and DNA-bound hetero-oligomeric complexes (down). Hydrogen bonds are shown as red dashed lines, and schematic diagrams illustrate the assembly with color-coded domains: DBD (DNA-binding domain), OD (oligomerization domain), and CTAD (C-terminal activation domain).

**(D)** Predicted Aligned Error (PAE) matrices for the structural models generated by AlphaFold3. The matrices assess the prediction quality and inter-chain confidence for the HSFA1 homotrimer (3×HSFA1, left), the HSFA2 homotrimer (3×HSFA2, middle), and the DNA-bound heterohexameric complex (3×HSFA1+3×HSFA2+E1-wild-probe, right). Dark green regions represent low expected position error, indicating high structural reliability for both intra-domain folding and inter-chain interaction interfaces.

**(E)** Dynamic interaction between HSFA1 and HSFA2 assessed by LCI under room temperature, heat stress, and recovery conditions.

**(F)** Transcriptional activity of the HSFA1–HSFA2 heterocomplex under room temperature, heat stress, and recovery conditions, measured as LUC/REN ratio in *N. benthamiana* leaves.

LUC/REN represents the ratio of firefly to *Renilla* luciferase activity. Data are the means ± SD (n = 3). Statistical significance was determined by Student's *t*-test (**P* < 0.05, ***P* < 0.01, ****P* < 0.001).

**Figure S14. Validation of additional interactions between RCA1 and chloroplast proteins.**

**(A)** Y2H assays examining the interactions between RCA1 and four additional candidate proteins (PsbY, VTE1, Lhcb5, Lhca3), in addition to the four interactions presented in the main figure.

**(B)** LCI assays examining the interactions between RCA1 and two additional candidate proteins (PsbY, VTE1), in addition to those presented in the main figure.

**(C)** Bimolecular fluorescence complementation (BiFC) assays of RCA1 with Lhca2, PetC, PsaK, RCA2, PsbY or VTE1 in *N. benthamiana* leaves. Reconstituted YFP signal (yellow) co-localizes with chloroplast autofluorescence (red), confirming that all interactions occur within the chloroplast. Empty vector combinations served as negative controls.

**Figure S15. Affinity purification of recombinant RCA1 and candidate interacting proteins.**

Affinity purification of RCA1 and candidate proteins. A: HIS-Lhca2; B: HIS-PetC; C: HIS-PsaK; D: HIS-RCA2; E: GST; F: GST-RCA1; M: 180 kDa protein marker; 1, 10, 19, 28, 37 and 46: filtrate; 2, 11, 20, 29, 38 and 47: column flow-through; 3, 12, 21, 30, 39 and 48: wash fraction; 4–9, 13–18, 22–27 and 31–36: imidazole gradient elution (each group: 40, 60, 80, 120, 160, 200 mM); 40–45 and 49–54: GSH gradient elution (each group: 5, 8, 10, 12, 15, 20 mM). Red arrows indicate proteins to be further concentrated by ultrafiltration. Candidate proteins that did not show interaction are not presented in the figure.

**Figure S16. Thermostability analysis of RCA1 and chloroplast proteins.**

Tm values of RCA1, Cpn60β, Lhca2, PetC, PsaK, and RCA2 as determined by DSF. Values with different letters are significantly different according to a one-way ANOVA followed by Duncan's test (*P* < 0.05).

**Figure S17. Phylogenetic tree of RCA genes across photosynthetic organisms.**

**(A)** Unrooted tree.

**(B)** Count of branches.

**Figure S18. Phylogenetic tree of RCA Clade II.**

Phylogenetic tree of *RCA* Clade II. γ represents the whole-genome triplication event. Red asterisks (*) indicate the duplication events in each lineage.

**Figure S19. Phylogenetic tree of RCA Clade I.**

Clade I_M, monocot branch. Clade I_E, eudicot branch.

**Figure S20. Motif analysis of RCA proteins.**

**(A)** Motif prediction using the CDD database.

**(B)** Motif prediction using MEME Suite.

The motifs with blue font color represent differences between Clade I and II.

**Figure S21. Tissue expression patterns of RCA genes in representative organisms.**

The relative expression values were standardized within 0 to 1.

**Figure S22. Expression patterns of RCA genes in response to abiotic stresses in representative plants.**

**Figure S23. Multiple sequence alignment of rice α-type RCA with other sequences.**

**Figure S24. Multiple sequence alignment of the α- and β-type RCA members in Panicoideae.**

**Figure S25. The working model of temperature-dependent transcriptional regulation of RCA1 by HSFA1 and HSFA2 in R. hainanense.**

Under room temperature, HSFA1 is constitutively expressed but remains inactive due to inhibition by HSP70/HSP90 chaperones, while HSFA2 and RCA1 expression is undetectable. Upon heat stress, HSFA1 is released from HSP70/HSP90 and functions as the master regulator of heat stress response, binding to heat shock elements to activate transcription of downstream targets including *HSFA2* and *HSP* genes. Both HSFA1 and newly synthesized HSFA2 independently activate *RCA1* expression, while their interaction forms a co-activation complex that further enhances *RCA1* transcription during sustained heat stress. Following return to room temperature, HSFA1 is re-sequestered by HSP70/HSP90, the HSFA1-HSFA2 interaction diminishes, and *HSFA2*/*RCA1* transcription ceases. Notably, residual HSFA2 protein persists and enables rapid reactivation of *RCA1* expression during subsequent heat stress through immediate interaction with HSFA1, demonstrating a characteristic heat memory response. Arrow thickness indicates relative expression levels.

**Table S1.** **Predicted potential interacting proteins of RCA1.**

| **Protein name** | **Protein ID** | **Amino Acid Number** | **Subcellular localization** | **Category (function/complex)** |
| --- | --- | --- | --- | --- |
| RhRCA2 | Rh_49176 | 467 | Chloroplast | Rubisco activase (carbon fixation regulation) |
| RhPsaK | Rh_80386 | 116 | Chloroplast | Photosystem I core protein (PSI complex) |
| RhPsbO | Rh_62227 | 331 | Chloroplast | Photosystem II core protein (PSII complex) |
| RhPsbS | Rh_67976 | 269 | Chloroplast | Photosystem II regulatory protein (non-photochemical quenching) |
| RhPsbY | Rh_77107 | 198 | Chloroplast | Photosystem II core protein (PSII complex) |
| RhLhca2 | Rh_50747 | 279 | Chloroplast | Light-harvesting complex protein (PSI-LHCI) |
| RhLhca3 | Rh_69411 | 271 | Chloroplast | Light-harvesting complex protein (PSI-LHCI) |
| RhLhcb4 | Rh_68697 | 296 | Chloroplast | Light-harvesting complex protein (PSII-LHCII) |
| RhLhcb5 | Rh_66186 | 286 | Chloroplast | Light-harvesting complex protein (PSII-LHCII) |
| RhLhcb6 | Rh_57083 | 259 | Chloroplast | Light-harvesting complex protein (PSII-LHCII) |
| RhPetC | Rh_73454 | 227 | Chloroplast | Electron transport protein (cytochrome b6f complex) |
| RhDEG1 | Rh_53519 | 433 | Chloroplast | Protein degradation enzyme (PSII repair and quality control) |
| RhSLC35 | Rh_51340 | 414 | Chloroplast | Protein degradation transporter (chloroplast protein metabolism) |
| RhCCT3 | Rh_40639 | 555 | Chloroplast | Molecular chaperone/foldase (protein degradation and assembly) |
| RhVTE1 | Rh_46339 | 516 | Chloroplast | Tocopherol synthase (antioxidant regulation) |

**Table S2. The RCA sequences used in phylogenetic tree.**

| **Species name** | **Gene ID** |
| --- | --- |
| *Actinidia chinensis* | Achn114341 |
| *Actinidia chinensis* | Achn240101 |
| *Actinidia chinensis* | Achn243801 |
| *Actinidia chinensis* | Achn257861 |
| *Actinidia chinensis* | Achn286671 |
| *Actinidia chinensis* | Achn336801 |
| *Actinidia chinensis* | Achn366371 |
| *Amborella trichopoda* | AmTrH2 08G034700 |
| *Amborella trichopoda* | AmTrH2 12G123800 |
| *Aquilegia coerulea* | Aqcoe1G103200 1 |
| *Aquilegia coerulea* | Aqcoe2G227300 1 |
| *Aquilegia coerulea* | Aqcoe6G179100 1 |
| *Arabidopsis thaliana* | AT2G39730 |
| *Arabidopsis thaliana* | AT1G73110 |
| *Brachypodium distachyon* | Bradi4g09120 |
| *Brachypodium distachyon* | Bradi4g09125 |
| *Brachypodium distachyon* | Bradi5g24590 |
| *Brassica rapa* | Brara C02027 |
| *Brassica rapa* | Brara D02409 |
| *Brassica rapa* | Brara E00605 |
| *Brassica rapa* | Brara G03289 |
| *Camelina sativa* | CsAcsn226 06G252700 |
| *Camelina sativa* | CsAcsn226 04G254100 |
| *Camelina sativa* | CsAcsn226 05G084000 |
| *Camelina sativa* | CsAcsn226 09G243000 |
| *Camelina sativa* | CsAcsn226 07G203100 |
| *Camelina sativa* | CsAcsn226 16G198500 |
| *Camellia sinensis* | CSS0014740 2 |
| *Camellia sinensis* | CSS0033329 5 |
| *Camellia sinensis* | CSS0049545 2 |
| *Camellia sinensis* | CSS0033885 1 |
| *Chlamydomonas reinhardtii* | Cre04 g229300 |
| *Chlamydomonas reinhardtii* | Cre17 g718950 |
| *Fragaria vesca* | gene18467 |
| *Fragaria vesca* | gene15880 |
| *Fragaria vesca* | gene26333 |
| *Fragaria vesca* | gene30208 |
| *Glycine max* | Glyma 02G249600 |
| *Glycine max* | Glyma 03G068100 |
| *Glycine max* | Glyma 11G221000 |
| *Glycine max* | Glyma 13G371900 |
| *Glycine max* | Glyma 14G067000 |
| *Glycine max* | Glyma 18G036400 |
| *Gossypium raimondii* | Gorai 010G200600 |
| *Gossypium raimondii* | Gorai 011G267600 |
| *Gossypium raimondii* | Gorai 012G106000 |
| *Gossypium raimondii* | Gorai 009G149200 |
| *Helianthus annuus* | HanXRQChr05g0136941 |
| *Helianthus annuus* | HanXRQChr09g0250121 |
| *Helianthus annuus* | HanXRQChr15g0496591 |
| *Helianthus annuus* | HanXRQChr07g0201601 |
| *Helianthus annuus* | HanXRQChr10g0312801 |
| *Helianthus annuus* | HanXRQChr08g0235911 |
| *Malus domestica* | MD01G1137900 |
| *Malus domestica* | MD03G1086300 |
| *Malus domestica* | MD04G1134100 |
| *Malus domestica* | MD07G1202200 |
| *Malus domestica* | MD11G1095300 |
| *Malus domestica* | MD12G1147900 |
| *Malus domestica* | MD09G1230800 |
| *Marchantia polymorpha* | Mapoly0022s0132 |
| *Marchantia polymorpha* | Mapoly0031s0065 |
| *Miscanthus sinensis* | MisinT073900 1 |
| *Miscanthus sinensis* | MisinT074000 1 |
| *Miscanthus sinensis* | Misin10G202500 1 |
| *Miscanthus sinensis* | Misin10G202700 1 |
| *Miscanthus sinensis* | Misin12G258100 1 |
| *Miscanthus sinensis* | Misin11G240300 1 |
| *Musa acuminata* | GSMUA_Achr10P00480_001 |
| *Musa acuminata* | GSMUA_Achr10P30850_001 |
| *Musa acuminata* | GSMUA_Achr11P24320_001 |
| *Musa acuminata* | GSMUA_Achr2P16450_001 |
| *Nymphaea colorata* | Nycol E00177 1 |
| *Nymphaea colorata* | Nycol F01224 1 |
| *Oryza sativa* | LOC_Os11g47970 |
| *Oryza sativa* | LOC_Os11g47980 |
| *Oryza sativa* | LOC_Os04g56320 |
| *Ostreococcus lucimarinus* | 3956 |
| *Ostreococcus lucimarinus* | 31184 |
| *Ostreococcus lucimarinus* | 37239 |
| *Physcomitrium patens* | Pp3c9_5280 |
| *Physcomitrium patens* | Pp3c9_5310 |
| *Physcomitrium patens* | Pp3c22_12960 |
| *Physcomitrium patens* | Pp3c15_12410 |
| *Physcomitrium patens* | Pp3c13_13170 |
| *Physcomitrium patens* | Pp3c4_22100 |
| *Picea abies* | MA_10433855g0010 |
| *Picea abies* | MA_99905g0010 |
| *Picea abies* | MA_379291g0010 |
| *Populus trichocarpa* | Potri 008G058500 |
| *Populus trichocarpa* | Potri 010G200500 |
| *Populus trichocarpa* | Potri 016G101100 |
| *Populus trichocarpa* | Potri 001G037300 |
| *Rhododendron delavayi* | Rhdel05G0043300 1 |
| *Rhododendron delavayi* | Rhdel05G0291800 1 |
| *Rhododendron delavayi* | Rhdel12G0232300 1 |
| *Rhododendron delavayi* | Rhdel13G0085400 1 |
| *Rhododendron griersonianum* | RHGRI_013185 |
| *Rhododendron griersonianum* | RHGRI_015292 |
| *Rhododendron griersonianum* | RHGRI_035110 |
| *Rhododendron griersonianum* | RHGRI_036302 |
| *Rhododendron irroratum* | 7740 1 |
| *Rhododendron irroratum* | 33570 1 |
| *Rhododendron irroratum* | 102630 1 |
| *Rhododendron irroratum* | 428110 1 |
| *Rhododendron molle* | Rm00G037090 2 |
| *Rhododendron molle* | Rm00G236000 2 |
| *Rhododendron molle* | Rm00G871520 5 |
| *Rhododendron ovatum* | Ro06157 2 |
| *Rhododendron ovatum* | Ro30609 1 |
| *Rhododendron ovatum* | Ro42837 1 |
| *Rhododendron ovatum* | Ro31300 1 |
| *Rhododendron simsii* | Rs00G144220 5 |
| *Rhododendron simsii* | Rs00G432960 2 |
| *Rhododendron simsii* | Rs00G735370 1 |
| *Saccharum officinarum* | Sh_203B14_p000060 |
| *Saccharum officinarum* | Sh_230B14_p000050 |
| *Saccharum officinarum* | Sh_211P07_p000010 |
| *Saccharum officinarum* | Sh_234F05_p000060 |
| *Selaginella moellendorffii* | 268623 |
| *Selaginella moellendorffii* | 79425 |
| *Selaginella moellendorffii* | 82367 |
| *Setaria italica* | Seita 3G023400 |
| *Setaria italica* | Seita 8G251200 |
| *Setaria italica* | Seita 8G251300 |
| *Setaria italica* | Seita 8G250400 |
| *Setaria viridis* | SEVIR_3G024200 |
| *Setaria viridis* | SEVIR_8G261466 |
| *Setaria viridis* | SEVIR_8G261433 |
| *Solanum lycopersicum* | Solyc09G000522 |
| *Solanum lycopersicum* | Solyc10G002940 |
| *Solanum lycopersicum* | Solyc03G003062 |
| *Solanum tuberosum* | Soltu DM 10G023180 |
| *Solanum tuberosum* | Soltu DM 10G020280 |
| *Solanum tuberosum* | Soltu DM 03G032070 |
| *Sorghum bicolor* | Sobic 005G231500 |
| *Sorghum bicolor* | Sobic 005G231600 |
| *Sorghum bicolor* | Sobic 006G248700 |
| *Spinacia oleracea* | Spov3_chr4 03364 |
| *Spinacia oleracea* | Spov3_chr5 02956 |
| *Theobroma cacao* | Thecc 10G038500 |
| *Theobroma cacao* | Thecc 05G034400 |
| *Theobroma cacao* | Thecc 06G132800 |
| *Thuja plicata* | Thupl 29379479s0005 1 |
| *Thuja plicata* | Thupl 29380247s0017 1 |
| *Thuja plicata* | Thupl 29379023s0006 1 |
| *Triticum aestivum* | TraesCS2A03G1167400 |
| *Triticum aestivum* | TraesCS2B03G1324900 |
| *Triticum aestivum* | TraesCS2D03G1117600 |
| *Triticum aestivum* | TraesCS4A03G0485000 |
| *Triticum aestivum* | TraesCS4A03G0484800 |
| *Triticum aestivum* | TraesCS4D03G0267500 |
| *Triticum aestivum* | TraesCS4D03G0267700 |
| *Triticum aestivum* | TraesCS4B03G0327500 |
| *Triticum aestivum* | TraesCS4B03G0327300 |
| *Vaccinium darrowii* | Vadar_g14666 t1 |
| *Vaccinium darrowii* | Vadar_g616 t1 |
| *Vaccinium darrowii* | Vadar_g619 t1 |
| *Vaccinium darrowii* | Vadar_g624 t1 |
| *Vaccinium darrowii* | Vadar_g617 t1 |
| *Vaccinium darrowii* | Vadar_g41714 t1 |
| *Vaccinium myrtillus* | Vmy03g6679 t1 |
| *Vaccinium myrtillus* | Vmy06g34205 t1 |
| *Vaccinium myrtillus* | Vmy09g23532 t1 |
| *Vaccinium myrtillus* | VmyS2524g14295 t1 |
| *Vitis vinifera* | VIT_206s0004g05180 1 |
| *Vitis vinifera* | VIT_208s0007g00840 2 |
| *Vitis vinifera* | VIT_213s0019g02050 4 |
| *Vitis vinifera* | VIT_209s0018g01270 1 |
| *Zea mays* | Zm00001d001995_T003 |
| *Zea mays* | Zm00001d048593_P001 |
| *Zea mays* | Zm00001d048592_T001 |

**Table S3. Primers used for vector reconstruction.**

| **Vectors** | **Primer name** | **Primer Sequences** |
| --- | --- | --- |
| pHB-YFP | YFP-RCA1-X1 | 5'-CTTGGATCCTCGAGCTGCAG AGATCCCAAACAGACCCTTGA-3' 5'-CCCTTGCTCACCATACTAGTG TTCAATGTATACAAGCAGCTTCC-3' |
| pHB-YFP | YFP-RCA1-X2 | 5'-CTTGGATCCTCGAGCTGCAG AGATCCCAAACAGACCCTTGA-3' 5'-CCCTTGCTCACCATACTAGTG GATGAAGGTGTCTGACCATAG-3' |
| pHB-YFP | YFP-RCA1-X4 | 5'-CTTGGATCCTCGAGCTGCAG AGATCCCAAACAGACCCTTGA-3' 5'-CCCTTGCTCACCATACTAGTG TTCAATGTATACAAGCAGCTTCC-3' |
| pHB-YFP | YFP-RCA1-X5 | 5'-CTTGGATCCTCGAGCTGCAG AGATCCCAAACAGACCCTTGA-3' 5'-CCCTTGCTCACCATACTAGTG AAGCTGGAGCTTACCAATAGAT-3' |
| pCAMBIA3301 | 35S::RCA1-X1 | 5'-GGGGGACTCTTGACCATGG AGATCCCAAACAGACCCTTGA-3' 5'-TTACCCTCAGATCTACCATGGT GTTCAATGTATACAAGCAGCTTCC-3' |
| pCAMBIA3301 | 35S::RCA1-X2 | 5'-GGGGGACTCTTGACCATGG AGATCCCAAACAGACCCTTGA-3' 5'-TTACCCTCAGATCTACCATGGT GGATGAAGGTGTCTGACCATAG-3' |
| pCAMBIA3301 | 35S::RCA1-X4 | 5'-GGGGGACTCTTGACCATGG AGATCCCAAACAGACCCTTGA-3' 5'-TTACCCTCAGATCTACCATGGT GTTCAATGTATACAAGCAGCTTCC-3' |
| pCAMBIA3301 | 35S::RCA1-X5 | 5'-GGGGGACTCTTGACCATGG AGATCCCAAACAGACCCTTGA-3' 5'-TTACCCTCAGATCTACCATGGT AAGCTGGAGCTTACCAATAGAT-3' |
| pCold TF | HIS-RCA1-X1 | 5'-CTCGAGGGATCCGAATTCTTCTTT TGGCAGATTCAAAGTCTTTGCAG-3' 5'-TCTAGACTGCAGGTCGAC CTAGTTCAATGTATACAAGCAGCTTCCATCAT-3' |
| pCold TF | HIS-RCA1-X2 | 5'-CTCGAGGGATCCGAATTCTTCTTT TGGCAGATTCAAAGTCTTTGCAG-3' 5'-TCTAGACTGCAGGTCGAC CTAGGATGAAGGTGTCTGACCATAGAAAGT-3' |
| pCold TF | HIS-RCA1-X4 | 5'-CTCGAGGGATCCGAATTCTTCTTT TGGCAGATTCAAAGTCTTTGCAG-3' 5'-TCTAGACTGCAGGTCGAC CTAGTTCAATGTATACAAGCAGCTTCCATCAT-3' |
| pCold TF | HIS-RCA1-X5 | 5'-CTCGAGGGATCCGAATTCTTCTTT TGGCAGATTCAAAGTCTTTGCAG-3' 5'-TCTAGACTGCAGGTCGAC TCAAAGCTGGAGCTTACCAATAGATTGC-3' |
| pTRV2 | TRV2-RCA1 | 5'-ATTCTGTGAGTAAGGTTACCGAATTC CTAGTGTTCGATGAGTCCGACG-3' 5'-TGAGCTCGGTACCGGATCC TTGGGTGGTCCCACCGA-3' |
| pCAMBIA1391-GUS | proRCA1::GUS | 5'-ACCTGCAGGCATGCAAGCTTC TACTACCAAGCACCTCCGC-3' 5'-TTACCCTCAGATCTACCATGG AGAAATCAAGGGTCTGTTTGGGA-3' |
| pGreenII0800-LUC | proRCA1-P1::LUC | 5'-GCTTGATATCGAATTCCTGCAG GAAAGAGAGGCACTGAGAG-3' 5'-GGATCCCCCGGGCTGCAG AGAAATCAAGGGTCTGTTTGGG-3' |
| pGreenII0800-LUC | proRCA1-P2::LUC | 5'-GCTTGATATCGAATTCCTGCAG CTACTACCAAGCACCTCCGC-3' 5'-GGATCCCCCGGGCTGCAG AGAAATCAAGGGTCTGTTTGGG-3' |
| pGreenII0800-LUC | proRCA1-P3::LUC | 5'-GCTTGATATCGAATTCCTGCAG TCACTCAAAGTTGAGGGTTCC-3' 5'-GGATCCCCCGGGCTGCAG AGAAATCAAGGGTCTGTTTGGG-3' |
| pGreenII62-SK | SK-HSFA2 | 5'-CGGCCGCTCTAGAACTAGTG ATGGAAGGAGTGACAGTGAAGG-3' 5'-CGAGGTCGACGGTATCGATA TCAAGGAACGTAAATCCTCAAACT-3' |
| pGreenII62-SK | SK-HSFA1 | 5'-CGGCCGCTCTAGAACTAGTG ATGGACACAGAGGACGGCG-3' 5'-CGAGGTCGACGGTATCGATA TCAGACTTTTTTAGGATCAGAACCA-3' |
| pCAMBIA1300-nLUC | nLUC-HSFA1 | 5'-GAGCTCGGTACCTCCGGATCC ATGGACACAGAGGACGGCG-3' 5'-GCGTACGAGATCTGGTCGAC GACTTTTTTAGGATCAGAACCAAGAG-3' |
| pCAMBIA1300-cLUC | cLUC-HSFA2 | 5'-GGGCGGTACCTCCGGATCC ATGGAAGGAGTGACAGTGAAGG-3' 5'-ACGAAAGCTCTGCAGGTCGAC TCAAGGAACGTAAATCCTCAAACT-3' |
| pGBKT7 | BD-HSFA2 | 5'-CATGGAGGCCGAATTC ATGGAAGGAGTGACAGTGAAGG-3' 5'-GCAGGTCGACGGATCC TCAAGGAACGTAAATCCTCAAACT-3' |
| pGBKT7 | BD-HSFA2ΔAHA | 5'-CATGGAGGCCGAATTC ATGGAAGGAGTGACAGTGAAGG-3' 5'-GCAGGTCGACGGATCC ACCACCATAATCCAAACCGGT-3' |
| pGBKT7 | BD-HSFA1 | 5'-CATGGAGGCCGAATTC ATGGACACAGAGGACGGCG-3' 5'-GCAGGTCGACGGATCC TCAGACTTTTTTAGGATCAGAACCA-3' |
| pGADT7 | AD-HSFA2 | 5'-GCCATGGAGGCCAGTGAATTC ATGGAAGGAGTGACAGTGAAGG-3' 5'-GATTCATCTGCAGCTCGAGCTC TCAAGGAACGTAAATCCTCAAACT-3' |
| pGADT7 | AD-HSFA1 | 5'-GCCATGGAGGCCAGTGAATTC ATGGACACAGAGGACGGCG-3' 5'-GATTCATCTGCAGCTCGAGCTC TCAGACTTTTTTAGGATCAGAACCA-3' |
| pAbAi | pAbAi-E1 | 5'-CGAACAATCTGGAAGAACAATCTG GAAGAACAATCTGGAAC-3' 5'-TCGAGTTCCAGATTGTTCTTCCAGATT GTTCTTCCAGATTGTTCGAGCT-3' |
| pAbAi | pAbAi-E4 | 5'-CGGGAGTTTCGGGAGTTTCGGGAGTT TCGGGAGTTTCGGGAGTTTCC-3' 5'-TCGAGGAAACTCCCGAAACTCCCGAAA CTCCCGAAACTCCCGAAACTCCCGAGCT-3' |
| pCold TF | HIS-HSFA2 | 5'-CTCGAGGGATCCGAATTC ATGGAAGGAGTGACAGTGAAGG-3' 5'-TCTAGACTGCAGGTCGAC TCAAGGAACGTAAATCCTCAAACTTCT-3' |
| pGEX-4T-1 | GST-HSFA1 | 5'-CCGCGTGGATCCCCGGAATTC ATGGACACAGAGGACGGCG-3' 5'-GTCACGATGCGGCCGCTCGAG TCAGACTTTTTTAGGATCAGAACCA-3' |
| pGADT7 | AD-RCA1 | 5'-GCCATGGAGGCCAGTGAATTC ATGGCGACTGCCGTTTCC-3' 5'-GATTCATCTGCAGCTCGAGCTC CTAGTTCAATGTATACAAGCAGCTTCCATC-3' |
| pGBKT7 | BD-Lhca2 | 5'-CATGGAGGCCGAATTC GCAGCTAATCCCGAAAGGCC-3' 5'-GCAGGTCGACGGATCCACGTT  TAGTTACACTGATTGTAGAATTGTGAGG-3' |
| pGBKT7 | BD-PetC | 5'-CATGGAGGCCGAATTC GCCACCAGTATTCCGGCG-3' 5'-GCAGGTCGACGGATCC AGACCACCATGGGTCTTCGC-3' |
| pGBKT7 | BD-PsaK | 5'-CATGGAGGCCGAATTC GATTTCATTGGCTCTCCCACAAATTTGAT-3' 5'-GCAGGTCGACGGATCC AATAGCACCCAGGTTCTTAAGGCC-3' |
| pGBKT7 | BD-RCA2 | 5'-CATGGAGGCCGAATTCGAAAAGTC  TGAAATCGATGAGAAGAAACAGACA-3' 5'-GCAGGTCGACGGATCC ATAAACGCAGCTCCCATTGTCAC-3' |
| pSPYNE-35S | nYFP-RCA1 | 5'-TGGCGCGCCACTAGTGGATCC ATGGCGACTGCCGTTTCC-3' 5'-AGCGGTACCCTCGAGGTCGAC GTTCAATGTATACAAGCAGCTTCCATCATC-3' |
| pSPYCE-35S | cYFP-Lhca2 | 5'-TGGCGCGCCACTAGTGGATCC ATGGCCTCCTCTGTTTGTGC-3' 5'-AGCGGTACCCTCGAGGTCGACACGTT  TAGTTACACTGATTGTAGAATTGTGAGG-3' |
| pSPYCE-35S | cYFP-PetC | 5'-TGGCGCGCCACTAGTGGATCC ATGGCTGCCTCTACTCTCTCC-3' 5'-AGCGGTACCCTCGAGGTCGAC AGACCACCATGGGTCTTCGC-3' |
| pSPYCE-35S | cYFP-PsaK | 5'-TGGCGCGCCACTAGTGGATCC ATGGCAACAACTTTGATGACTACTCTC-3' 5'-AGCGGTACCCTCGAGGTCGAC AATAGCACCCAGGTTCTTAAGGCC-3' |
| pSPYCE-35S | cYFP-RCA2 | 5'-TGGCGCGCCACTAGTGGATCC ATGGCTTCAACCATCTCCACCG-3' 5'-AGCGGTACCCTCGAGGTCGAC ATAAACGCAGCTCCCATTGTCAC-3' |
| pGEX-4T-1 | GST-RCA1 | 5'-CCGCGTGGATCCCCGGAATTC ATGGCGACTGCCGTTTCC-3' 5'-GTCACGATGCGGCCGCTCGAG CTAGTTCAATGTATACAAGCAGCTTCCATCAT-3' |
| pCold TF | HIS-Lhca2 | 5'-CTCGAGGGATCCGAATTC ATGGCCTCCTCTGTTTGTGCT-3' 5'-TCTAGACTGCAGGTCGACTCAACGTT  TAGTTACACTGATTGTAGAATTGTGAG-3' |
| pCold TF | HIS-PetC | 5'-CTCGAGGGATCCGAATTC ATGGCTGCCTCTACTCTCTCC-3' 5'-TCTAGACTGCAGGTCGAC TTAAGACCACCATGGGTCTTCGC-3' |
| pCold TF | HIS-PsaK | 5'-CTCGAGGGATCCGAATTC ATGGCAACAACTTTGATGACTACTCTCC-3' 5'-TCTAGACTGCAGGTCGAC TCAAATAGCACCCAGGTTCTTAAGGC-3' |
| pCold TF | HIS-RCA2 | 5'-CTCGAGGGATCCGAATTC ATGGCTTCAACCATCTCCACCG-3' 5'-TCTAGACTGCAGGTCGAC TTAATAAACGCAGCTCCCATTGTCACTC-3' |
| pESC-TRP | pESC-RCA1 | 5'-AGGAGAAAAAACCCCGGATCC ATGGCATCAACTTTCATTACCATGTCTTC-3' 5'-CAACTTCTGTTCCATGTCGACATGATGATGA  TGATGATGAGAACCACCACCACCGTAACCGTA  TCCTGAATTGTCCATGGG-3' |
| pESC-TRP | pESC-Lhca2 | 5'-AGGAGAAAAAACCCCGGATCC ATGGCCTCCTCTGTTTGTGCT-3' 5'-CAACTTCTGTTCCATGTCGACATGATGATGATGATGA  TGAGAACCACCACCACCACGTTTAGTTACACTGATTG  TAGAATTGTGAGG-3' |
| pESC-TRP | pESC-PetC | 5'-AGGAGAAAAAACCCCGGATCC ATGGCTGCCTCTACTCTCTCC-3' 5'-CAACTTCTGTTCCATGTCGACATGATGATGATGATGA  TGAGAACCACCACCACCAGACCACCATGGGTCTTCGC-3' |
| pESC-TRP | pESC-PsaK | 5'-AGGAGAAAAAACCCCGGATCC ATGGCAACAACTTTGATGACTACTCTCC-3' 5'-CAACTTCTGTTCCATGTCGACATGATGATGATGATGA  TGAGAACCACCACCACCAATAGCACCCAGGTTCTTAAGGCC-3' |
| pESC-TRP | pESC-RCA2 | 5'-AGGAGAAAAAACCCCGGATCC ATGGCTTCAACCATCTCCACCG-3' 5'-CAACTTCTGTTCCATGTCGACATGATGATGATGATGA  TGAGAACCACCACCACCATAAACGCAGCTCCCATTGTCAC-3' |

**Table S4. Quantitative RT-PCR primers.**

| **Gene name** | **Primer Sequences** |
| --- | --- |
| *18S rRNA* | 5'-CGCATTCCCCACTGTATTAGAC-3' 5'-CGTAACAAGGTTTCCGTAGGTG-3' |
| *AtActin2* | 5'-TCCCTCAGCACATTCCAGCAGAT-3' 5'-AACGATTCCTGGACCTGCCTCATC-3' |
| *GUS* | 5'-TACCGACGAAAACGGCAAGA-3' 5'-CGGTGATATCGTCCACCCAG-3' |
| *RCA1-X1* | 5'-ACGTTGTATGCGCCCCTAAT-3' 5'-GAGCTGCTTTGCCATAGAAAGTT-3' |
| *RCA1-X2* | 5'-GACAAGCTCCTCGAGTACGG-3' 5'-TGCTTTGCCTCCTGCTAAGG-3' |
| *RCA1-X3* | 5'-GTACCTAAGCGAAGCAGCCC-3' 5'-TCTCAGGCTTTGTATCAGCCA-3' |
| *RCA1-X4* | 5'-TGTGGTCACTGGTATTTTCAGGA-3' 5'-CCTTAGAGCGCCGAAGAAGT-3' |
| *RCA1-X5* | 5'-CGGATTGGTGTTTGCACAGG-3' 5'-TTTCATCATCTGGAGCGCCT-3' |
| *RCA2* | 5'-AGAACAGGAGAATGTGAAGAGA-3' 5'-TGAGCTGCTTGACCATAGAA-3' |
| *RCA3* | 5'-GACGGCTAGCAAGTACCTGTG-3' 5'-AAAGAGCAAGTGTCATCCCTCC-3' |
| *HSFA2* | 5'-CTCGCGAAAGCCCTACATTG-3' 5'-CCCAACTCTTCCTCCTGGTTC-3' |
| *HSFA1* | 5'-CGATTCCGAACGCAAATGCT-3' 5'-TTCCACACGACGAAGCTGTT-3' |
| *AtRCA* | 5'-TCGCACAGAGCAACAAGAAGAG-3' 5'-TCATCAGAAGTGTCGTAGGCAAGA-3' |
| *RCA1-rt* | 5'-AGTGTTCGATGAGTCCGACG-3' 5'-CCGAATCCATGGGAGCTTGA-3' |
